# Triclosan: A Small Molecule with Controversial Roles

**DOI:** 10.3390/antibiotics11060735

**Published:** 2022-05-30

**Authors:** Maria Stefania Sinicropi, Domenico Iacopetta, Jessica Ceramella, Alessia Catalano, Annaluisa Mariconda, Michele Pellegrino, Carmela Saturnino, Pasquale Longo, Stefano Aquaro

**Affiliations:** 1 Department of Pharmacy, Health and Nutritional Sciences, University of Calabria, 87036 Arcavacata di Rende, Italy; s.sinicropi@unical.it (M.S.S.); domenico.iacopetta@unical.it (D.I.); jessica.ceramella@unical.it (J.C.); michele.pellegrino@unical.it (M.P.); stefano.aquaro@unical.it (S.A.); 2 Department of Pharmacy-Drug Sciences, University of Bari Aldo Moro, 70126 Bari, Italy; 3 Department of Science, University of Basilicata, 85100 Potenza, Italy; annaluisa.mariconda@unibas.it (A.M.); carmela.saturnino@unibas.it (C.S.); 4 Department of Chemistry and Biology, University of Salerno, Via Giovanni Paolo II 132, 84084 Fisciano, Italy; plongo@unisa.it

**Keywords:** antimicrobials, triclosan, TCS, endocrine disrupting chemicals, personal care products, Colgate Total^®^, toxicity

## Abstract

Triclosan (TCS), a broad-spectrum antimicrobial agent, has been widely used in personal care products, medical products, plastic cutting boards, and food storage containers. Colgate Total^®^ toothpaste, containing 10 mM TCS, is effective in controlling biofilm formation and maintaining gingival health. Given its broad usage, TCS is present ubiquitously in the environment. Given its strong lipophilicity and accumulation ability in organisms, it is potentially harmful to biohealth. Several reports suggest the toxicity of this compound, which is inserted in the class of endocrine disrupting chemicals (EDCs). In September 2016, TCS was banned by the U.S. Food and Drug Administration (FDA) and the European Union in soap products. Despite these problems, its application in personal care products within certain limits is still allowed. Today, it is still unclear whether TCS is truly toxic to mammals and the adverse effects of continuous, long-term, and low concentration exposure remain unknown. Indeed, some recent reports suggest the use of TCS as a repositioned drug for cancer treatment and cutaneous leishmaniasis. In this scenario it is necessary to investigate the advantages and disadvantages of TCS, to understand whether its use is advisable or not. This review intends to highlight the pros and cons that are associated with the use of TCS in humans.

## 1. Introduction

**Figure 1:**
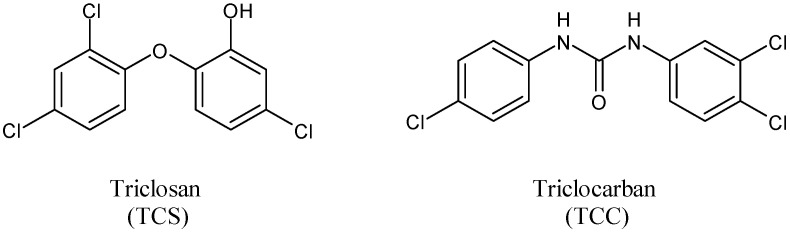
Triclosan and triclocarban.

Triclosan (TCS), along with its congener triclocarban (TCC) (Figure 1) [1], is one of the most widely used antimicrobial ingredients in several pharmaceuticals and personal care products (PPCPs), such as toothpastes, facial cleansers, deodorants, soap bars, textiles, medical devices, and plastic cutting boards [2]. It was also found in contaminated food, such as seafoods [3]. TCS, namely 5-chloro-2-(2,4-dichlorophenoxy)phenol or 2,4,4′-trichloro-2′-hydroxy-diphenyl ether, is an ether whereas TCC belongs to the diarylureas family [4,5]. Despite their different chemical structures, TCS and TCC are often mentioned together as these compounds are small molecules sharing three chlorine atoms in their structures [6].

**Figure 2:**
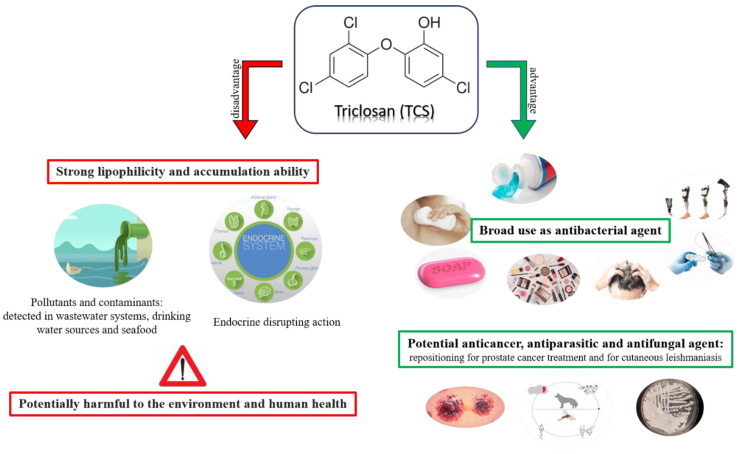
Advantages and disadvantages of Triclosan (TCS).

TCS (CAS#3380-34-5), also named Irgasan DP300, CH3565, GP41-353, FAT 80′023, Irgacare MP and Irgasan [7], was first licensed for use in 1964 by the Swiss company Ciba-Geigy and has rapidly become a ubiquitous substance to human exposure. Since then, TCS has been used as an antimicrobial primarily in personal care products (Figure 2) [8]. Over 80% of TCS usage is contributed to cosmetics, personal care products, and household cleaning products which contain mostly between 0.1 and 0.3% of TCS [9]. Colgate Total^®^ toothpaste, containing 10 mM TCS, was the number two leading toothpaste in sales in 2016 [10] with an 11.8% increase in sales from 2015 to 2016 [11]. In toothpastes, TCS has demonstrated an important action in reducing plaque and gingivitis [12,13]. TCS may be also found, as a material preservative, in a vast range of consumer products including kitchen utensils, clothes, fabrics, etc. [14,15]. Moreover, TCS-coated sutures have shown a significant reduction of the risk of surgical site infections (SSIs) during surgical interventions [16], even though this finding is almost controversial [17].

The worldwide production of TCS is very high. It has been recently estimated that it reaches 1500 tons per year and that 132 million liters of TCS-containing products are used annually in the USA alone [18]. The production rates have increased since the outbreak of the novel coronavirus disease 2019 (COVID-19) [19] due to the high demand for disinfection [20,21]. The injudicious use of disinfectants, including TCS, may promote the development of antimicrobial resistance [22,23], potentially the most far-ranging global health challenge following the COVID-19 pandemic [24]. TCS was also found in food, such as tuna from Indonesia, Nile perch from Lake Victoria, farmed pangasius and shrimp from Asia, mackerel from Spain, plaice, mackerel and mussels from The Netherlands, and mussels from the North Sea and the Mediterranean Sea. Among European people, Spanish adults, followed by Portuguese and Italian adults, had the highest exposure to bisphenol A, methylparaben, and TCS through their seafood diet [25]. The bacteriostatic action of TCS prevents microbes from growing at low concentrations and is due to the inhibition of an enzyme that is involved in fatty acid synthesis, the enoyl-acyl carrier reductase (ENR). Thus, TCS is classified among fatty acid synthase inhibitors (FASNs) [26]. Moreover, TCS directly kills microbes at higher concentrations by destabilizing bacterial membranes and by introducing intercalating defects into a bacterial membrane (SCCS 2010), exerting a bactericidal activity [27]. TCS is also active against bacterial biofilms [28,29]. However, the greatest concern over the potential health effects of TCS is related to its endocrine disrupting action, supporting the demand of a total ban on the use in everyday products [30]. TCS belongs to endocrine disrupting chemicals (EDCs) which are the substances that change the course of endocrine systems in a way that adversely affects the organism itself or its progeny [31,32]. It is classified among emerging pollutants (Eps), also known as contaminants of emerging concern (CECs) [33]. Significant concentrations of TCS have been detected in different aquatic organisms [34]. The main absorption pathways for TCS are skin and oral absorption [35]. TCS was found to adversely affect the hypothalamic-pituitary-thyroid axis in both experimental studies using rodents and observational studies of humans [36]. In women of reproductive age, exposure to TCS is of particular concern given the ability of TCS to cross the placenta and fetal blood-brain barrier and enter breast milk [37]. Exposure to TCS has been reported to cause early menarche among the overweight or obese [38], and to play a role in allergen and food sensitization [39]. Moreover, TCS exposure has been associated with neurodevelopment impairment, metabolic disorders, and cardiotoxicity [40]. Recent studies describe the potential immunotoxicity of TCS in humans, often leading to allergies [41]. Studies regarding the role of TCS in cancer are almost controversial. Some papers report that the TCS increases the risk of cancer [42], whereas other articles suggest TCS for repositioning in treating cancer, particularly prostate cancer [43,44]. Actually, the growing resistance of the wide groups of bacteria, the toxicity that is exhibited on different aquatic organisms, the adverse health effects that are observed in vitro and in vivo, and the available epidemiological studies suggest that further efforts to monitor TCS toxicity at environmental levels are necessary. Monitoring TCS and its occurrence in aquatic environments have been widely described with analytical methods including chromatography-mass spectrometry, electrochemistry, capillary zone electrophoresis, and spectrophotometry [45]. Biomonitoring studies indicate nearly universal TCS exposure among pregnant women and children [46,47]. In September 2016, the U.S. Food and Drug Administration (FDA) developed a regulation, effective in 2017, establishing that certain active ingredients, including TCS, TCC, and 17 other antimicrobial chemicals, used in over-the-counter (OTC) consumer antiseptic products that are intended for use with water “are not generally recognized as safe and effective (GRAS/GRAE) and are misbranded” [48]. Following an evaluation of TCS by the Biocidal Products Committee of the European Chemicals Agency (ECHA), the European Commission (EC) decided in 2016 that TCS is not approved for use in human hygiene biocidal products [49,50], but maintained its legality as a preservative in selective cosmetics and mouthwashes in concentrations up to 0.3% and 0.2%, respectively [18,51]. Since the beginning of February 2017, TCS was no longer available in such products in the E.U. However, from some reports, TCS application in personal care products is still allowed [52]. The U.S. National Institute of Environmental Health Sciences (NIEHS) and Environmental Protection Agency (EPA) declare TCS as an EDC (NIEHS 2020) [53], while according to the European Food Safety Authority (EFSA) and European Chemical Agency (ECHA), it is still under assessment as EDCs (ECHA 2020) [54]. TCS has been widely used as an antimicrobial additive in food storage containers and kitchen utensils, commonly marketed under the trademark Microban^®^ [55,56]. In those household articles, TCS is generally incorporated directly into the polymer matrix before curing in concentrations up to 1% (*w/w*) [57,58]. TCS is also marketed under the trade name Microban^®^ when it is used in plastics and clothing, and Biofresh^®^ when it is used in acrylic fibers [59]. Microban^®^ has been also reported to be used to coat door handles in hospitals [60]. Given its toxicity, TCS was removed from the list of authorized additives in materials in contact with food in the European Union in 2010 [61] (European Commission, 2010). This action only applies to companies and products that are manufactured within the EU. However, TCS is still present in several antimicrobial food containers that are purchased through e-commerce and its migration rates to food can be very high. In addition, microplastics containing TCS were found to leach from the inner surface of food containers when they were exposed to either oven or microwave heating [62]. Given the numerous advantages and disadvantages that are related to the use of TCS, in this review we want to describe a roundup of both studies, so that each reader may be able to independently evaluate whether it is convenient to continue using TCS. Moreover, in order to better understand the pharmacokinetic activities that are related to TCS, also ADME (absorption, distribution, metabolism, and excretion) properties, have been addressed.

## 2. Pharmacological Activities of TCS

### 2.1. Antimicrobial Activity

TCS has shown diverse activities as an antimicrobial agent; indeed, several reports describe its antibacterial and antifungal activity, also against biofilms [63,64]. TCS has demonstrated high biocidal and durable activity on polyester and cotton surfaces for medical textile applications [65]. To a lesser extent, triclosan is used in textiles and plastics (sportswear, bed clothes, shoes, carpets) to control the growth of disease or odor-causing bacteria [66]. Recently, the use of TCS in self-disinfecting paints has been considered as a promising strategy towards cleaner indoor environments by preventing the colonization of microorganisms on the surface of walls [67].

#### 2.1.1. Antibacterial and Antifungal Activity

TCS is an effective antimicrobial against both Gram-positive and Gram-negative bacteria [68,69] and fungi [70]. The use of TCS in cutting boards may provide a hygienic barrier only under certain conditions (low humidity, long exposure time, and clean conditions) but not against all genera of bacteria. The study of Møretrø et al. (2011) demonstrated that at a relative humidity of 100%, no antibacterial effect of the TCS-containing board was found against *Escherichia coli*, *Salmonella*, *Staphylococcus aureus*, and *Serratia* spp. The only exception was *Listeria monocytogenes* [71]. A recent study on the canal system of primary teeth demonstrated that the combination of TCS with eugenol is more effective in comparison to amoxicillin and eugenol against *Enterococcus faecalis* [72], a most frequent human nosocomial pathogen [73]. TCS exposure has been shown to reduce the susceptibility to clinical antimicrobials, such as ciprofloxacin and levofloxacin, in *E. coli* isolates from urine samples [74]. Multiple studies have demonstrated an increase of the minimum inhibitory concentration (MIC) of TCS from approximately 4- to 60-fold for specific bacteria, which is related to the interaction of TCS with different genes, for example *ycjD* and *fabI* genes for *E. coli* and *fabV* gene for the Gram-negative *Pseudomonas aeruginosa* [75]. Recently, eugenol-loaded TCS microemulsions have been suggested for use in place of conventional biocidal solutions in endodontic therapy where complete elimination of microorganisms from the treatment site is required [76]. Recently, a solution containing 0.15% TCS was studied for the treatment of denture stomatitis affecting complete denture wearers. This treatment promoted denture stomatitis remission and a microbial load decrease of Candida spp., especially *Candida albicans*, as well as the Gram-negative microorganisms, *Staphylococcus* spp. and *S. mutans*, of the intaglio surface of denture [77].

#### 2.1.2. Antibiofilm Activity

TCS has shown interesting activity against biofilms [63]. The study by Jongsma et al. (2015) [78] analyzed the biofilm accumulation on the wires that are permanently bonded to the anterior teeth used after orthodontic treatment to prevent the teeth from relapsing to pre-treatment positions. The use of antibacterial toothpastes marginally reduced the amount of biofilm on the wires, but significantly reduced the viability of the biofilm organisms. Major shifts in the biofilm composition were obtained by combining a TCS-containing toothpaste (Colgate Total^®^; Colgate-Palmolive Company, Piscataway, NJ, USA) with an essential oil-containing mouth-rinse. The use of antibacterial toothpastes without the mouth-rinse lowered the viability of adhering bacteria *Lactobacilli*, *Streptococcus sobrinus* ATCC 33478, and *S. mutans* ATCC 10449. It was noticed that the combination of TCS-containing toothpaste with an essential oil-containing mouth-rinse yielded a reduction in the prevalence of *S. mutans* from 30% to 5%, determining a drastic shift in the composition of the oral microbiome. However, the TCS-containing toothpaste alone produced major increases in the prevalence of adherent *S. oralis*/*S. mitis*, *S. sanguinis,* and *S. mutans*. Thus, the authors hypothesize that the non-polar TCS could increase the hydrophobicity of the *S. mutans* cell surface, facilitating the removal of *S. mutans* by hydrophobic oils, such as oil-containing mouth-rinses [78]. In a recent work, it has been demonstrated that TCS may deplete the membrane potential in *Pseudomonas aeruginosa* biofilms [79]. This action leads to the inhibition of the adaptive resistance, a phenotypic response that allows *P. aeruginosa* to transiently survive aminoglycosides including as tobramycin. TCS was shown to enhance tobramycin effectiveness in vivo in a mouse wound model. The association of TCS with tobramycin may be considered a new antibiofilm strategy as it enhances the susceptibility of *P. aeruginosa* biofilms to aminoglycosides [80]. The combination of TCS with ethylenediaminetetraacetic acid (EDTA) and cranberry were significantly effective in eradicating and preventing biofilm formation of *Escherichia coli* strains on Foley catheters [81]. Antiseptic mouthwashes for personal oral hygiene are widely used for their ability to inhibit dental plaque, a complex biofilm that is formed in a series of discrete steps. Plaque begins to form with the accumulation of Gram-positive streptococci, followed by increasing deposits, which involve Gram-negative anaerobic bacteria. A recent study on a mouth rinse spray containing 0.03% TCS, Plax^®^, showed that the growth of *S. mutans* is inhibited by this mouth rinse on toothbrush bristles that were used by children [82].

#### 2.1.3. Antiparasitic Activity

In vitro and ex vivo drug assays on promastigotes and amastigotes showed that TCS possesses antileishmanial activity against *Leishmania donovani* with a half minimal inhibitory concentration (IC_50_) of 30 µM. Studies in silico suggested that this action was due to the interaction of TCS with *L. donovani* enoyl-acyl carrier protein reductase [83]. TCS has been studied for its activity as an antitubercular drug. It has been shown to suppress mycobacterial growth at low concentrations by directly inhibiting InhA, an essential ENR, resulting in the lysis of *Mycobacterium tuberculosis* while at higher concentrations it determined the disruption of bacterial protein synthesis. It is noteworthy that, although less active than the antitubercular drug isoniazide, TCS doesn’t require any activation and is able to directly affect the function of InhA [84]. Several studies demonstrated the activity of TCS in the treatment of malaria against two different targets. TCS specifically targets both wild-type and pyrimethamine-resistant *Plasmodium falciparum* and *P. vivax* dihydrofolate reductases, classic targets for the blood stage of the parasite. It inhibits blood-stage *Plasmodium*, via its action against dihydrofolate reductase, and the liver stage of the parasite, via the inhibition of ENR [85]. Several recent studies are aimed at obtaining new analogs of TCS that are more active and less toxic than the parent compound [86,87].

## 3. Studies on Toothpastes Containing TCS

Toothpastes containing TCS have been shown to reduce plaque and gingivitis [88,89]. Most studies on toothpastes containing TCS are related to periodontitis, a highly prevalent, chronic, non-specific, and immunologically devastating disease of periodontal tissues, caused by microbial infection. A toothpaste containing TCS and fluoride compared to toothpaste with fluoride, reduces plaque, gingivitis, and bleeding, but did not show a significant effect on clinical attachment loss [90,91]. A marketed dentifrice control (Colgate Total^®^, Colgate-Palmolive, New York, NY, USA) contains 0.3% TCS along with 0.24% sodium fluoride [10,91,92]. A recent study compared the effect of 0.3% TCS, 2.0% the polyvinylmethyl ether maleic acid (PVM/MA) copolymer + 1450 ppm fluoride (test group) and 1450 ppm fluoride (control group) [93]. It was demonstrated that the toothpaste containing 0.3% TCS was more effective than the regular fluoride toothpaste in improving the periodontal clinical condition around natural teeth of periodontally healthy subjects, presenting at least one implant that had been treated for peri-implantitis, enrolled in a regular maintenance program for two years. They received a regular adult soft bristles toothbrush (Colgate Palmolive, São Paulo, Brazil) as well as dental floss (Colgate Palmolive, São Paulo, Brazil) and interdental toothbrushes (Colgate Palmolive, São Paulo, Brazil). TCS toothpastes reduced clinical attachment and bleeding on probing. Another study compared three commercial dentifrice formulations, one of which contained TCS (sodium fluoride/TCS/copolymer) and other two (stannous fluoride/sodium hexametaphosphate/zinc lactate and sodium fluoride) in 35 adults. The study showed that the sodium fluoride/TCS/copolymer toothpaste consistently determined significant reductions for a range of microorganisms (specifically six microbial types, anaerobes, *Streptococci*, *Actinomyces*, hydrogen-sulphide (H_2_S)-producing bacteria, *Fusobacteria,* and *Veillonella*) in diverse oral sites in comparison with the other two dentifrice formulations as seen 12 h after brushing [94]. In another work, the use of a fluoride dentifrice containing TCS and the PVM/MA copolymer in Colgate Total^®^ toothpaste determined a greater uptake of TCS to enamel and buccal epithelial cells than from a dentifrice containing TCS alone. Moreover, Colgate Total^®^ provided superior protection against plaque and gingivitis, caries, oral malodor, exhibited superior stain removal, and provided protection against the progression of periodontal disease [95]. Another study was carried out in smokers [96]. Tobacco smoking is an established risk factor for periodontitis and is associated with periodontal attachment and tooth loss. A TCS/copolymer/fluoride-containing dentifrice (test; Totals, Colgate; Piscataway, NJ, USA), was compared with a fluoride-containing dentifrice (control; Double Cool Stripes, Colgate; Piscataway, NJ, USA) that did not contain the TCS/copolymer. The results suggest that an oral hygiene regimen including a TCS/copolymer/fluoride dentifrice may sustain the short-term effect of non-surgical periodontal therapy in smokers. A recent study on rats demonstrated the therapeutic effectiveness of TCS and an anti-inflammatory flurbiprofen-loaded nanogels system in ligature-induced experimental periodontitis in rats [97]. TCS activity in periodontitis is related to the activation of human periodontal ligament fibroblasts that are induced by lipopolysaccharide from *Porphyromonas gingivalis* [98]. Recently, the link between TCS and chitosan, a natural polymer that may act as a drug delivery agent and exerts antibacterial and anti-inflammatory activities [99], has been investigated in the prevention and/or treatment of inflammation in periodontal diseases. Chitosan-TCS particles were able to modulate the inflammatory response in gingival fibroblasts [100].

## 4. Absorption, Distribution, Metabolism, and Excretion (ADME) Properties and Photodegradation

Exposure to TCS may occur mainly through oral intake and dermal contact [101]. Moreover, exposure to TCS via air, surface water, drinking water, and soil may also occur, leading to the long-term exposure [102]. TCS can be absorbed through the oral mucous membrane within one to four hours [103]. After oral ingestion of TCS, a rapid gastrointestinal absorption and median urinary excretion of 54% may occur within four days [104]. The second main exposure route to TCS is dermal exposure, with an absorption that is lower than 10% [105]. The halogenated biphenyl ether structure of TCS is stable to hydrolysis (in the pH range 4–9) [45]. TCS is a nonvolatile compound and has a moderate water solubility (12 mg/L). The phenolic moiety in the TCS structure gives weak acidic properties (p*K*_a_ = 8.1 at 20 °C). The high octanol-water Partition coefficient (log K_ow_ 4.76) and high hydrophobicity leads to bioaccumulation potential [106]. The half-life for TCS was reported to range from 1.3–1.4 d in water to 53.7–60.3 d in sediments [107]. Recently, a great variability in the half-life and degradation kinetics has been demonstrated depending on the nature of medium, availability of light, initial concentration, and other factors. For example, in drinking water it has been reported to be 41 min, in salt water 4 d, in fresh water 8 d, and in water sediment from 120 to 540 d [34]. As an organochloride compound, TCS is sensitive to photodegradation and its photolytic half-life has been reported to vary from 41 min in sunlight to 39.8–55.99 d in darkness [108]. TCS is predominately excreted in urine as a glucuronide or sulfate conjugate [104]. The short urinary half-life of 9 to 32 h makes it an excellent indicator of recent exposure rather than long-term exposure [109]. A study by Dix-Cooper et al. (2019) [110] in Asian immigrant women living in Vancouver (Canada) showed that a 12-fold increase in urinary TCS concentrations was observed among Colgate Total^®^ toothpaste users compared to non-users.

### 4.1. Metabolism and Transformation Products of TCS

**Figure 3:**
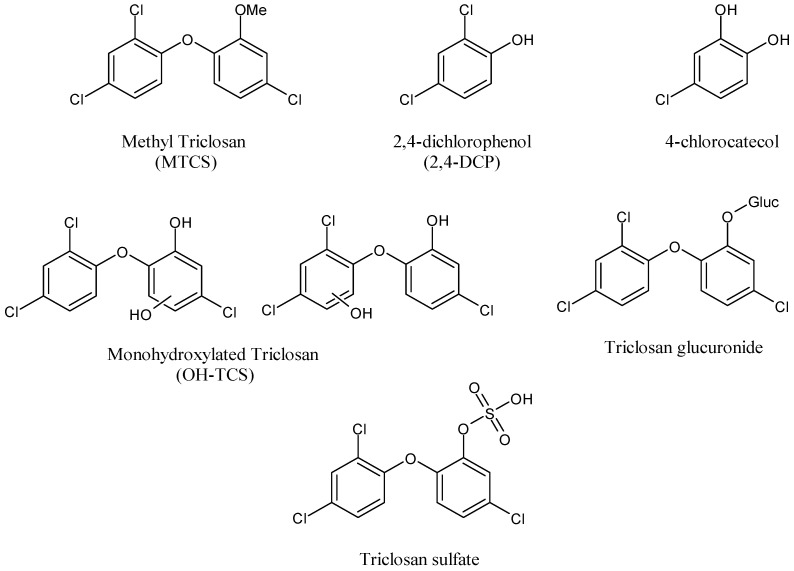
Structures of TCS transformation products.

TCS is transformed biologically into several compounds (Figure 3), including methyltriclosan (MTCS) [111], which can be formed during aerobic treatment; 2,4-dichlorophenol (2,4-DCP); and 4-chlorocatechol [112,113].

Similar to the parent compound, TCS transformation products have themselves demonstrated undesirable environmental and health characteristics. MTCS has been demonstrated to be more persistent than TCS and, similar to TCS, possesses endocrine disrupting capabilities [114,115]. TCS is excreted in the feces and urine [116]. Rats and mice show predominantly biliary excretion into the feces, whereas guinea pigs excrete the majority of the dose via the kidney. In humans, urinary excretion is the major route of elimination, with fecal elimination being the secondary route. TCS is metabolized via Phase I and Phase II reactions. Cytochrome P450 leads to monohydroxylated TCS derivatives, whereas Phase II reactions occur via UDP-glucuronosyltransferases (UGTs) and sulfotransferases leading to TCS glucuronide and TCS sulphate, respectively. The conjugation of TCS in the presence of human liver microsomes or cytosol and in skin was demonstrated in vitro [117]. Recently, TCS hydroxylated metabolites (monohydroxylated TCS) were detected in human stool, while other conjugates of OH-TCS, namely OH-TCSG and OH-TCSS deriving respectively from glucuronidation and sulfation reactions occurred on OH-TCS, were identified in the urine samples. It was also demonstrated that excretions of hydroxylated metabolites are isomer-specific and conjugate-selective [118]. TCS has a chemical structure that is similar to dioxin.

### 4.2. Photodegradation of TCS

**Figure 4:**
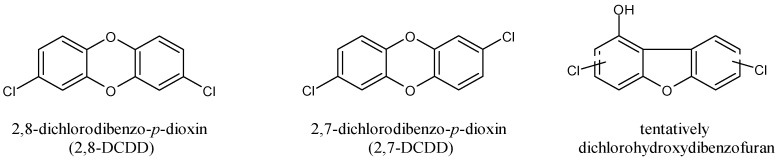
Products deriving from photodegradation of TCS.

In photochemical reactions and incineration processes TCS could be converted to the more toxic compound 2,8-dichlorodibenzo-*p*-dioxin (2,8-DCDD) [119]. Photodegradation appears to be one of the major routes of elimination of TCS in aquatic environments and takes place at a low light intensity under ultraviolet (UV) light (254, 313, or 365 nm), simulated solar light, or artificial white light. Figure 4 shows the products deriving from TCS photodecomposition. Recently, it was demonstrated that Fe and Mn oxides mediated the transformation of TCS into 2,8-DCDD without sunlight at room temperature under near dry conditions in the natural environment [120]. The photochemical formation of dichlorodibenzodioxin from TCS in both solid phase and thin films of TCS has been demonstrated. Moreover, highly toxic photoproducts, including 2,8-DCDD, 2,4-DCP, and possibly dichlorohydroxydibenzofuran derivatives, were identified in water samples (Figure 4). The photodegradation of TCS and formation of 2,8-DCDD occur over a wide range of pH levels (3.0–9.0), with the rate of formation being faster at a basic pH. The formation of 2,7-dichlorodibenzo-*p*-dioxin (2,7-DCDD) has been also suggested [117]. There are three other dioxin congeners, 2,3,7-TCDD, 1,2,8-TriCDD, and 1,2,3,8-TCDD, that have been also described as photoproducts of chlorinated derivatives of TCS [121].

## 5. Toxicity Studies on TCS

Despite the numerous and several years of studies about TCS, it remains a controversial antibacterial regarding its toxicity. Rodrigues et al. (2007) [122] compared the genotoxicity of one antiseptic that is currently used for odontologic treatment containing TCS (Plax^®^) and other two, containing cetylpyridinium chloride and chlorhexidine digluconate (Cepacol^®^ and Periogard^®^, respectively) by the Somatic Mutation and Recombination Test in *Drosophila melanogaster* [123], employing flies having normal bioactivation (the standard cross) and flies with increased cytochrome P450-dependent biotransformation capacity (the high bioactivation cross). Plax produced negative responses in both types of flies, as well as Periogard, while Cepacol produced positive responses in both the assays. The recent study by Querido et al. (2022) [67] demonstrated that a self-disinfecting paint containing TCS showed low levels of cyto- and genotoxicity, which were evaluated by direct contact and on extracts that were obtained from leaching following ISO 10993 and by comet assay and cytokinesis-block micronucleus assay, respectively. According to the REACH (Registration, Evaluation, Authorisation and Restriction of Chemicals) criteria for persistence and bioaccumulation, TCS would classify as a non-persistent and nonbioaccumulative [124]. However, most studies describe TCS as a toxic agent. Below, some other studies regarding the potential toxicity of TCS are described. Moreover, recent studies attribute the toxicity of TCS to its metabolites. The mesocosm study that was carried out by Contardo-Jara et al. (2021) [30] to investigate the fate and bioaccumulative potential of TCS and its main transformation product MTCS in water and sediment demonstrates that MTCS is bioaccumulative in contrast to its parent. Li (2021) showed that the chlorination of TCS caused a 30-fold increase of antiestrogenic activity [125].

### 5.1. Effects on Hypothalamic-Pituitary-Thyroid Axis and Steroidogenesis

TCS is often considered a potential thyroid disruptor. However, its hormone disruptive potential has mainly been studied in animals and requires confirmation in human studies [126]. The adverse effects of TCS were evaluated by applying the Navigation Guide systematic review methodology on three studies on humans and eight studies that were conducted in rats. The authors concluded that there was “sufficient” non-human evidence and “inadequate” human evidence of an association between TCS exposure and thyroxine concentrations, and thus TCS is “possibly toxic” to reproductive and developmental health [127]. TCS may reduce thyroxine concentrations by activating nuclear receptors to increase hepatic catabolism of thyroxine [128]. The results from epidemiological studies of TCS and thyroid function in humans are not consistent [129,130]. A recent study on young male Wistar albino rats showed that a high dose of TCS led to symptoms of hypothyroidism that were expressed by significant changes in FT3, FT4, and TSH (triiodothyronine, thyroxine, and thyroid-stimulating hormone, respectively) levels [131]. The same reduction of FT3 and FT4 was observed in Sprague-Dawley rats [132] and female mice [133]. The effects on thyroid hormones luteinizing hormone (LH) and follicle-stimulating hormone (FSH) were studied in vivo in female mice. The results depended on doses and dosing regimens. The administration of TCS in concentrations up to 0.345 mmol/kg/day determined a decrease of LH and FSH [133], while at the higher oral dose (0.639 mmol/kg/day), an increase of both LH and FSH was detected in Sprague-Dawley rats [134]. TCS exposure has been also related to diminished ovarian reserve. A study on about 500 females aged between 25 and 39 years showed that TCS exposure may negatively affect antral follicle count, a marker of ovarian reserve, whereas no statistically significant associations between other parameters of ovarian reserve (estradiol, FSH, and AMH levels) and TCS concentrations [135]. Steroidogenesis is an important target of TCS. Studies on primary rat granulosa cells (rGCs) that were treated with TCS demonstrated that it increased estradiol, progesterone production, and up-regulated the ovarian steroidogenesis pathway [136]. TCS increased estradiol and progesterone levels with upregulated steroidogenesis gene expression at concentrations ranging from 0 to 10 µM [137]. Basini et al. (2021) [138] recently explored the effect of different concentrations of TCS on cultured luteal cells that were isolated from swine ovaries. TCS was shown to interfere with the main function of the luteal cells thus suggesting that it can disrupt the physiological function of the corpus luteum, a transient endocrine organ that is essential for a correct ovarian cyclicity and for a successful pregnancy.

### 5.2. Effects on Semen

The endocrine system is important for male reproductive development because androgens, such as testosterone, promote the maturation of male secondary characteristics and the process of spermatogenesis. Male reproductive health, specifically testosterone and sperm count were declined, which is correlated with an increase in a variety of EDCs [139]. In vitro studies showed that TCS binds to androgen receptors and exhibits an antiandrogenic activity in human breast cancer cells [140]. This chemical reduces testosterone production by disrupting cholesterol biosynthesis in Leydig cells [141]. TCS decreases the weights of the testes and sex accessory organs, leading to a decrease in sperm density [142]. TCS has also exhibited a tendency to accumulate in the epididymis [143]. In rats, TCS has shown antiandrogenic effects and detrimentally affects reproductive functions and fertility of males. Priyanka et al. (2019) found that gestational and lactational exposure to TCS impaired the reproduction and fertility, changed testicular physiology and functions, and repressed the testosterone levels of F1 male rats [144]. Ha et al. (2018) found that TCS suppressed testicular steroidogenesis via the miR-6321/JNK/Nur77 cascade [145]. Recently, the same research group demonstrated that TCS-induced miR-142-5p inhibits P450c17 by the JAK1/STAT1 pathway and downstream Sp1/DNMT1/DAX1 cascade, finally facilitating testosterone suppression [146]. In a cross-sectional investigational study, the association between TCS exposure that was measured by urinary TCS concentration and semen quality in men that were recruited from male reproductive health clinics was evaluated. TCS was shown to affect human sperm production and normal morphology in humans, as studied in 471 men [147]. A successive study on 315 men provided evidence that exposure to TCS is associated with poorer semen quality [148]. In the paper by Nassan et al. (2019) [149], 262 men were enrolled in the Environmental and Reproductive Health (EARTH) between 2009 and 2017. Urinary TCS concentrations were classified into four quartiles where the lowest quartile included all concentrations that were below the limit of detection that ranged between 1 and 2.3 μg/L. Consistent patterns of lower percent morphologically normal sperm were found for men with urinary TCS in the second or third quartile compared to undetectable concentrations. This association was stronger for samples that were obtained prior to 2013 when TCS was more often detectable in urine [149]. However, recent studies suggest TCS is not a hormone-disrupting chemical. Specifically, a study on male Wistar rats demonstrated the absence of effects of TCS in the Hershberger assay, as well as on the parameters that were evaluated in the reproductive toxicity study [150]. Another work on serum testosterone levels in human adult males found that TCS did not reduce testosterone levels [151]; low-dose TCS exposure was not associated with risks of abnormal semen quality in a study on 406 men from a reproductive clinic [152]. In a study on children and adolescents aged 6–19 years, little association of TCS and testosterone levels was found [153].

### 5.3. Studies on Prenatal Exposure to TCS

TCS is suspected of having endocrine-disrupting properties, but few human studies have examined the developmental effects of prenatal TCS exposure. Some epidemiological studies report that gestational urinary TCS concentrations during pregnancy are associated with decreased birth weight and length, head circumference, and gestational age [154,155]. Moreover, TCS exposure has been related to spontaneous abortion [156], gestational diabetes [157], and decrease fecundity [158]. Prenatal TCS exposure in Chinese pregnant women was associated with increased cord testosterone levels and decreased placental steroidogenic enzyme levels, namely human cytochrome P450-family19-subfamily A-polypeptide 1 (*CYP19A1* or *P450arom*), human 3 beta hydroxysteroid dehydrogenase type 1 (*3β-HSD*), and human 17 beta hydroxysteroid dehydrogenase type 1 (*17β-HSD*) [159]. There are contradictory studies regarding the association between gestational TCS exposure and birth weight: Zhong et al. (2020) [160] concluded that there is no association, while the conclusion of Khoshhali et al. (2020) [161] suggested that gestational TCS exposure was associated with greater birth weight. However, none of the two studies applied the Navigation Guide criteria to their evaluations that are recommended for evaluating evidence streams within environmental health. A systematic review and meta-analysis [162] of 15 studies have been recently carried out, which makes use of a random effects model, estimating differences in these outcomes per 10-fold increase in TCS concentrations and considering TCS levels and infant sex as sources of heterogeneity. It makes use of the Navigation Guide Methods, thus evaluating the risk of bias within individual studies and across the body of evidence. The available epidemiological evidence of moderate quality and low risk of bias provides limited evidence that gestational TCS exposure is associated with reductions in infant birth weight. Differences in this association across studies may be related to the level of TCS exposure in source populations. Moreover, in a major part of the considered studies, TCS exposure was found to be higher in North America and Europe compared to Asia. The study by Lassen et al. showed that prenatal TCS exposure was associated with reduced anogenital distance (AGD) at three months of age in boys [163], although this is not consistent between studies [164,165,166]. A recent study showed that the exposure to TCS during gestation in mice led to cognition dysfunction and impairments in sociability and social novelty preference, impaired acquisition of spatial learning and reference memory in offspring that were derived from dams that were exposed to TCS, deficits in nesting behavior, and increased anxiety-like behavior, without significant change in depression-like behaviors [167]. A recent systematic review and meta-analysis indicated that exposure to TCS during pregnancy has no significant influence on maternal levels of thyroid hormone [168]. Prenatal exposure to TCS is also related to differences in maternal FT4 and TSH concentrations. Specifically, Aker et al. suggested that prenatal exposure to TCS may be associated with an increase of maternal FT4 and TSH [169]. Wang et al. (2017) also found a positive association between TCS exposure during pregnancy and maternal FT4 levels [170]. Berger et al. (2018) found that TCS was associated with lower maternal total FT4 levels during pregnancy [171]. However, several other studies found no relationship between TCS and thyroid hormone levels in pregnant women [172,173]. Recently, Li et al. (2022) demonstrated that exposure to TCS is associated with abnormal placenta growth and fetal development during pregnancy in mice. Moreover, the authors found that TCS caused placenta dysfunction that was characterized by the significant reduction in weight and size of the placenta and fetus by PPARγ inhibition or silence [174]. A recent study during early pregnancy demonstrated that TCS is not associated with thyroid function [175]. A recent study from the Health Outcomes and Measures of the Environment (HOME), on a prospective pregnancy and birth cohort in Ohio, considered the health effects that are associated with early-life exposure to TCS. Urinary TCS concentrations at delivery, but not during mid- to late-pregnancy and childhood, were inversely associated with child intelligence quotient at age eight years of age in the cohort of U.S. children that were studied [176]. A successive study by the same group showed that increasing gestational and childhood urinary TCS concentrations were associated with higher behavior problem scores in eight year old boys, but not girls [177].

### 5.4. Studies on the Effects of TCS during Lactation

A recent study was carried out to evaluate the influence of TCS on the male reproductive system of postnatal pups. Pregnant female Wistar rats (*Rattus norvegicus*) and their male offspring were studied. The lactating mother rats were dosed with 0 mg, 3 mg, and 5 mg/kg/day of TCS until 28 days after the day of delivery so that the pups consumed TCS through breastfeeding. It was noticed that the mothers passed the detrimental effects to their untreated male pups as shown by reduced androgen synthesis and subsequently decreased sperm count [178].

### 5.5. Neurotoxic and Hepatotoxic Effects of TCS

Exposure to TCS may adversely affect neurodevelopment and may exert adverse effects on central nervous system (CNS) functions, mainly through the induction of apoptosis and oxidative stress [179]. Neurotoxicity was demonstrated in vitro by Szychowski et al. (2016) [180] and Park et al. (2016) [181], in mouse neocortical neurons and neural stem cells, respectively. The chronic exposure of TCS on experimental zebrafish (*Danio rerio*) embryos highlighted that TCS caused heart edema and slow heartbeat [182], delayed hatching and increased mortality [183], and impaired lipid metabolism [184] at concentrations up to 1.25 mg/L. A recent study on neural development using *Danio rerio* embryos confirmed that the exposure to TCS during neurodevelopment, especially during axonogenesis, is toxic and suggests a possible mechanism, through which TCS-induced ectopic expression of proneural genes may affect sequential neural development and impair glial cell function, resulting in CNS structural abnormalities during neurogenesis such as reduced axon growth and synaptic density [185]. In vivo studies that were obtained by exposure to TCS (0.3 and 0.6 mg/L) of adult zebrafish for 48 h showed that it induced anxiety-like behavior by reducing acetylcholinesterase (AChE) activity in the brain. Moreover, erratic movements that were probably related to AChE inhibition in skeletal muscle, were observed [186]. Successive studies on zebrafish larvae demonstrated that TCS is a neurotoxic agent even at sublethal concentrations [187]. A recent study showed that chronic TCS exposure reduced social dominance in adult mice, by inducing ultrastructural damage to hippocampal neurons and synapses in adult mice and impaired memory formation in female mice [188]. TCS has been also related to liver injury. It disrupts normal liver functioning and development in embryonic zebrafish and induces hepatotoxicity [189]. Some of these toxic events may be related to the involvement of gut microbiota.

### 5.6. Metabolic Disorders, Nephrotoxicity, and Polycystic Ovary Syndrome (PCOS)

Several researches have revealed to some extent that TCS exposure could induce nephrotoxicity. TCS administration induced function alterations, oxidative stress, pro-inflammatory, and fibrotic response in mouse kidneys. The lipid accumulation related to lipotoxicity and the decreased fatty acid oxidation may play a causative role in TCS-induced nephrotoxicity [190]. TCS has been related to metabolic disorders such as obesity and Type 2 diabetes [191,192]. The relationship between TCS and body mass index (BMI) using National Health and Nutrition Examination Surveys (NHANES) was investigated between 2003 and 2008: TCS exposure is associated with increased BMI [193]. TCS exposure is also associated with decreased bone mass density and increased osteoporosis [194]. Recently, exposure to TCS and TCC has been associated with increased risk of childhood obesity, by examining in first morning urine of children aged 7–11 years by liquid chromatography coupled to mass spectrometry [195]. In another study in children, a relationship between TCS and its metabolite MTCS with predictive indicators of cardiovascular diseases and obesity was found [196]. A recent study on a total of 674 infertile Chinese women at 18–45 years of age showed that TCS exposure at a relatively low level was associated with PCOS [197].

### 5.7. Immune Response, Asthma and Allergies

Over the last decade, researchers have shown that endocrine disrupting chemicals, such as TCS, can affect the development, function, and lifespan of immune cells. TCS may attenuate immunity against infections or cause a hyperactivity of immune responses such as allergies and autoimmune diseases. Exposure to TCS has been related to food and aeroallergy and asthma exacerbation in humans, both in adults [198] and children [199]. Although TCS did not directly act as a sensitizer, it was demonstrated to increase the allergic response in a mouse model of asthma on SKH1 mice. It was shown that repeated dermal exposure to TCS alter the skin barrier integrity and microbiome, thus probably contributing to the increase in allergic immune responses after dermal exposure to TCS [200]. In another study on striped catfish *Pangasianodon hypophthalmus*, TCS induced immunosuppression and reduced the survivability of this fish during the challenge to a fish pathogenic bacterium *Edwardsiella tarda* [201]. The topical skin application of TCS has demonstrated to be sufficient to induce peanut sensitivity in mice [202]. The associations between allergic diseases and TCS has been recently studied in Taiwanese children. TCS levels were positively correlated with serum IgE levels and significantly associated with asthma, allergic rhinitis, and atopic dermatitis among boys [203].

## 6. Gut Microbiota and Microbiome Involvement in TCS Exposure

The understanding that humans readily absorb TCS and are able to pass TCS through breastmilk has led to a need to explore the impact of TCS on the human gut microbiome. Moreover, the extensive use of TCS over the past several decades [204], along with the increase in inflammatory bowel disease [205] in youth populations around the world, has raised concerns over how TCS and other antimicrobials may impact the gut microbial community [206]. Sanidad et al. (2019) asserts that TCS could have adverse effects on the gut microbiome and gut health [207], but the impact of TCS exposure through household and personal care products on the developing microbiota is unknown. Thus, several recent papers are addressed to the study of the disturbance of gut microbiota by TCS. TCS exposure disturbs the gut microbiota in various animal model systems, including fathead minnows [208], zebrafish [209], rats [210], and mice [211]. The perinatal exposure of rats to TCS (50 mg/kg/day, i.e., the lowest toxic oral dose in rats) led to disturbances of the metabolism and gut microbiota that were long-lasting and persisted even after the exposure had been terminated [212]. TCS was also suggested to disturb the gut microbiome in humans. Bever et al. (2018) reported that the gut microbiomes of infants who received breast milk containing TCS had significantly lower alpha diversity with respect to the infants who received breast milk with non-detectable levels of TCS. Moreover, the relative abundances of certain bacteria were also modulated in the infants who were fed TCS-containing breast milk [213]. A randomized study of TCS-containing household and personal care products that was carried out during the first year following birth showed that TCS exposure did not induce global reconstruction or loss of microbial diversity. The routine usage of TCS-containing toothpastes enhanced the relative abundance of broadly antibiotic-resistant species from the phylum *Proteobacteria* in adults, as well as in infants with high urinal concentrations of TCS [214]. A recent study, by means of the SHIME^®^ (simulator of the human intestinal microbial ecosystem) system, was used to simulate the human gut microbial community in vitro, and to look at the community in the three different regions of the gut, the ascending colon, transverse colon, and descending colon regions, as well as in both the lumenal and mucosal phases. It was found that treatment with TCS significantly impacted the community structure in terms of reduced population, diversity, and metabolite production, most notably in the ascending colon region. However, after a two-week recovery period, most of the population levels, community structure, and diversity levels were recovered for all the colon regions [215]. The involvement of the gut microbiota is also related to colitis and colon cancer. A brief exposure to TCS at relatively low doses, has caused low-grade colonic inflammation, an increase in colitis, and exacerbates colitis-associated colon cancer in mice probably via gut microbiome-dependent mechanisms. Moreover, these effects in vivo are partially dependent on Toll-like receptor 4 (TLR4) activation [216]. Finally, the involvement of gut microbiota and TCS has been studied in TCS-induced liver injury. The results showed that the disease was induced by gut microbiota via the gut–liver axis [217]. Interestingly, probiotics were used to modulate the microbiota and palliate intestinal metabolic disorders due to TCS exposure in animal models. Particularly, *Lactobacillus plantarum* ST-III increases the diversity of the gut microbiota in zebrafish, thus lowering the toxicity of chronic exposure to TCS. Additionally, a probiotic-rich diet reduced the risk of lipid-metabolism disorders such as increased triglyceride and total cholesterol levels [218].

## 7. Mechanism of Action in Pharmacological and Toxicity Studies

Several mechanisms of action have been proposed for TCS, most of which are related to the different effects, either positive or toxic, that are exerted by this compound [219]. TCS at low concentrations acting as a FASN inhibitor, i.e., it inhibits the synthesis of fatty acids, which are essential for the composition of bacterial membranes and cell wall; at high concentrations, TCS directly disrupts bacterial membranes [220]. TCS antimicrobial activity against certain types of fungi and various types of bacteria may be related to the block of the active site of the ENR, which is an essential enzyme for fatty acid biosynthesis in fungi and bacteria. This enzyme is required for fatty acid and biotin biosynthesis [221] and is not present in humans [222]. Maiden et al. (2020) [80] suggested that, in addition to its canonical mechanism inhibiting membrane biosynthesis, TCS can exert antibacterial properties by functioning as a protonophore that targets *P. aeruginosa* energetics. At consumer-relevant, non-cytotoxic doses, TCS inhibits the functions of both mitochondria and mast cells, a ubiquitous cell type [223]. Recently, the involvement of calcium in the mechanism of action of TCS has been suggested. TCS inhibits Ca^2+^ dynamics in mast cells [224], and it has been recently demonstrated that is also inhibits Ca^2+^ mobilization in human Jurkat T-cells [225]. The mechanisms of neurodevelopmental impairment occurring after TCS exposure may be connected to reactive oxygen species (ROS) activation and apoptosis induction. TCS induced ROS overproduction which ultimately leads to oxidative stress with a loss of membrane integrity, membrane depolarization, photosynthesis inhibition, and mitochondrial membrane depolarization. TCS induced an increase in caspase 3/7 activity and altered the expression of metacaspase genes which are indicative of apoptosis [226]. Szychowski et al. (2016) [180] demonstrated that TCS induces aryl hydrocarbon receptor (AhR)-dependent apoptosis in mouse neocortical neurons in vitro. The authors postulated that TCS exhibits primary and secondary effects. The primary effects such as impairment of Cyp1a1 signaling are mediated by TCS-induced ROS production, whereas the secondary effects of TCS are due to the transcriptional activity of AhR and estrogenic properties of TCS. Recent studies associate the neurotoxicity of TCS to AChE activity both by direct binding and by indirectly inducing increased oxidative stress [227]. Kim et al. (2018) [185] demonstrated that TCS increased apoptosis in the CNS and particularly affected the structure of the CNS, resulting in decreased synaptic density and shortened axon length in a study on neural development using zebrafish models. In addition, TCS significantly up-regulated the expression of genes that are related to axon extension and synapse formation such as a1-Tubulin and Gap43, while decreasing Gfap and Mbp expression related to axon guidance, myelination, and maintenance. Immunotoxicity may be related to miR-19a regulation. miR-19a is up-regulated by RNA-seq under TCS exposure. miR-19a up-regulation triggered the down-regulation of its target gene *socs3b*, and simultaneously activated the downstream IL-6/STAT3 signaling pathway [228]. The hepatic damage due to TCS exposure is also related to gut microbiota dysbiosis. Zhang et al. (2022) [217] recently demonstrated that TCS dramatically disturbed gut microbiota homeostasis, resulting in the overproduction of lipopolysaccharides (LPS) and deficiency of secondary bile acids such as deoxycholic acid and lithocholic acid. In addition, TCS considerably increased intestinal permeability by reducing mucus excretion and expression of tight junction proteins (ZO-1, occluding, and claudin 4), which facilitated the translocation of LPS. The LPS accumulation in blood contributed to liver injury by triggering the inflammatory response via the TLR4 pathway. The pulmonary toxicity of TCS is not clear. A study in rats that were exposed to single intratracheal instillation of TCS may induce acute inflammation in the lungs and affect the lung function ability by increasing the level of total cells count, polymorphonuclear leukocytes, total protein (TP), lactate acid dehydrogenase, tumor necrosis factor alpha (TNF-α), and interleukin-6 (IL-6) in bronchoalveolar lavage fluid (BALF) at one day after instillation. However, most pulmonary toxicity marker levels except TP in BALF was restored 14 days after instillation [229]. A study in Sprague–Dawley rats that were submitted to nasal inhalation exposure showed that the no-observed-adverse-effect concentration in rats was determined to be 0.13 mg/L. There were no treatment-related effects in rats of either sex below this concentration. At 0.40 mg/L, rats showed an enhancement in the incidence of postdosing salivation and a decrease in body weight [230]. Besides toxicity after absorption of TCS, dermal toxicity has to be considered. A combined metabolomic and lipidomic approach was used in human HaCaT keratinocytes to investigate the dermal toxicity that was triggered by TCS at a metabolic level. TCS exposure determined the increase of purine and glutathione (GHS) metabolism, the down-regulation of amino acid metabolism, and the dysregulation of lipid metabolism in keratinocytes, thus leading to overproduction of reactive oxygen species (ROS) and accumulation of ammonia. A total of 35 small molecular metabolites and 49 lipids were identified with significant changes in human keratinocytes [231]. The role and mechanisms of TCS in endothelial cells (ECs) have been recently investigated on human umbilical vein endothelial cell (HUVEC) function. The treatment with TCS suppressed HUVECs viability, migration, and angiogenesis, and enhanced the expression of inflammatory markers and ROS in HUVEC cells, at least in part via the PI3K/Akt/mTOR signaling pathway [232]. Spontaneous abortion has been also related to a decline of estrogen sulfotransferase activity both in mice and humans [156].

### 7.1. Toxicity Studies in Animal Models

In this subparagraph, studies regarding the possible mechanisms of toxicity in animal models are summarized. Yoon et al. (2017) showed that TCS induces oxidative stress and intracellular accumulation of toxins, at least in part, by disrupting the SKN-1/Nrf2-mediated oxidative stress response, specifically by inhibiting the nuclear localization of SKN-1/Nrf2 and the expression of its target genes, in both a model organism, the nematode *Caenorhabditis elegans* and human mesenchymal stem cells (hMSCs) [233]. A recent study on mosquitofish (*Gambusia affinis*) that were exposed to TCS showed that the up-regulations *Sirt1* and *Sirt2* in Sirtuin family could activate the Nrf2/ARE signaling pathway and the down-regulations of *miR-34b* and the up-regulations of *miR-34a*, *miR-144,* and *miR153a-5p* modulated the Nrf2/ARE signaling pathway [234]. Sublethal concentrations of TCS enhanced glutamic oxaloacetic transaminase (GOT), glutamic pyruvic transaminase (GPT), and glutathione-S-transferase enzyme activity in all the tissues with an increased dose and exposure period in the gill, liver, and muscle of an Indian major carp *Catla catla* [235]. Studies on the fly *Drosophila melanogaster* showed that TCS caused a redox imbalance with an increase on the catalase activity and a decrease on reactive oxygen species (ROS) level [236]. Liu et al. (2019) indicated that TCS exposure induces apoptosis of hepatocytes through the activation of the MAPK/p53 signaling pathway [237]. Studies in the larvae of *Labeo rohita* showed that TCS caused biochemical and transcriptomic alterations that resulted in oxidative stress, an impairment of metabolic processes, and dysfunction of the liver, kidney, and digestive system. A total of 96 h exposure to 0.06–0.097 mg/L TCS significantly declined the levels of glucose, triglycerides, urea, and uric acid and the activity of alkaline phosphatase, GOT and GPT [238].

### 7.2. Cytotoxicity Studies

Although TCS is considered to be an EDCs, it is not considered a carcinogen by the International Agency for Research on Cancer (IARC) [7]. Indeed, recent studies suggest its use as an anticancer agent. Actually, TCS has been proposed for repositioning as a preventive and anticancer agent, specifically for prostate cancer, as detailed in the Section 9. However, some in vitro and in vivo recent studies report its activity as cancerogenic in ovary, liver, prostate, colon, breast, and oral cancers. Specifically, an in vitro assay demonstrated that TCS can amplify the growth of BG-1 ovary cancer cells by regulating the expressions of cell cycle- and apoptosis-related genes via ER-dependent pathways [239]. In addition, chronic dietary exposure to TCS produced an increased incidence of liver tumors in mice, even though human studies are lacking in number. The mechanism for liver tumor induction has been attributed to the activation of either peroxisome proliferator activated receptor α (PPARα) or constitutive androstane receptor [240]. A recent study in mice demonstrated that TCS treatment enhances sorafenib resistance in hepatocellular carcinoma (HCC) cells, suggesting that exposure of TCS is detrimental to HCC patients during chemotherapy. Specifically, TCS induced the expression of drug-resistance genes in a highly aggressive HCC cell line, namely MHCC97-H cells, accelerates the clearance of sorafenib, and attenuates the antiproliferation effect of sorafenib. TCS decreased the antitumor effect of sorafenib on subcutaneous and intrahepatic growth of MHCC97-H in nude mice [241]. A study on LNCaP prostate cancer cells demonstrated that TCS enhanced LNCaP cell proliferative activity and migration. The mechanism that is proposed for cancerogenic activity of TCS is that it may enhance the progression of prostate cancer by regulating cell cycle- and metastasis-related genes via the AR signaling pathway [242]. Sanidad et al. (2019) [207] speculated that TCS exposure exaggerates colonic inflammation and colon cancer. Moreover, TCS has been suggested to promote breast cancer progression via an ER-mediated signaling cascade [243]. TCS induced MCF-7 breast cancer cell line proliferation via nongenomic ER signaling pathway that is associated with IGF-1R. TCS promoted the cell viability of MCF-7 cells via estrogen receptor α (ERα) similar to the positive control, 17β-estradiol (E2). In the same study, TCS was shown to induce tumor growth also in an in vivo xenografted mouse model [244]. In another study, TCS induced breast cancer progression via the stimulation of cell proliferation, anti-ROS production, anti-ER stress response, and anti-apoptosis by regulating the expression of ROS-induced ER stress-associated genes, including p-eIF2α, CHOP, Bcl-xl, and Bax [245]. In a recent study, the effects of TCS on cell migration and invasion have been evaluated on three human breast epithelial cell lines, namely the immortalized but non-transformed MCF-10F breast epithelial cells, the estrogen-responsive MCF-7 breast cancer cells, and the estrogen-unresponsive MDA-MB-231 breast cancer cells, using concentrations that are comparable with those in human tissues. Long-term exposure to 10^−7^ M of TCS resulted in increased migration and invasion as measured by xCELLigence technology for all three cell lines. The authors suggested a mechanism involving epithelial to-mesenchymal transition (EMT), as the reduction in levels of E-cadherin mRNA and of E-cadherin protein was observed [246]. A recent study in a within the Multiethnic Cohort (MEC) of postmenopausal women with breast cancer showed that breast cancer risk was weakly inversely associated with TCS exposures, mainly among overweight/obese women [247]. Finally, TCS may affect the level of DNA methylation in the human oral squamous carcinoma cell line (SCC-15) exerting a toxic effect only in the highest concentrations of 50 and 100 µM. TCS influences SOD activity in the SCC-15 cell line after 24 and 48 h treatment and decreases the mRNA expression of *DNMT3A* and *DNMT3B* in the SCC-15 cell line after 24 h treatment [248]. Summing up, several studies agree with the potential role of TCS in promoting cancer onset and progression and, unfortunately, contributing to the resistance phenomena, in vitro and in vivo models.

## 8. Removal from Aquatic Environment/Degradation Techniques

Due to the potential ecological and adverse health effects of TCS, several strategies are addressed to find new technologies for specific and effective removal of TCS from water, thus minimizing its threats in the environment. TCS mitigation from environmental matrices was obtained by using various oxidoreductases, including laccases, and peroxidases including soybean peroxidase, versatile manganese peroxidase, and horseradish peroxidase [249]. Photo-degradation techniques have been widely applied to the degradation of TCS. UV radiation at a basic pH or the presence of a catalyst may enhance TCS photodegradation, whereas TCS photodegradation is inhibited at an acidic pH or under the presence of scavengers [250]. Recently, a novel biological degradation method for the removal of TCS in municipal wastewater has been proposed, which includes the identification of degrading bacteria from municipal wastewater sludge. It was found that bacterial strain belonging to *Providencia rettgeri* group, namely *P. rettgeri* MB-IIT strain, could be advantageously used to degrade TCS that was present in the wastewater [251]. A recent study on the plant *Glyceria maxima* showed that the amounts of TCS in plant shoots were 1.4–2.5 times higher than that in roots [252]. Li (2021) [125] reported that ozonation of TCS mitigated the toxicity of the treated water. Moreover, several TCS adsorption methods have been reported [253]. A recent study proposed an alternative treatment using low-cost adsorbents, soybean hulls, and açaí seeds that were functionalized with iron oxide (Fe_3_O_4_) nanoparticles [254].

## 9. Repositioning of TCS

The drug repositioning process significantly saves the development time of new drugs. It only costs researchers approximately 3–12 years to develop an old drug for new treatments compared to the time required to develop a new drug that is approximately 12–17 years [255]. Repositioning has been suggested for numerous purposes, in diseases such as cancers [256,257] and the recent COVID pandemic [19]. TCS, as well as other FASN inhibitors, has shown to inhibit cancer cell growth by inducing cell death. TCS was found to be a superior alternative to C75, the synthetic derivative of antifungal agent cerulenin, and orlistat in triggering cell death in PCa cells via the inhibition of FASN. In addition, it induced G0/G1 cell cycle arrest and dose-dependent reduction in the total lipid content of PCa cells [258]. As a result, the TCS-mediated suppression of the metabolic oncogene FASN was suggested as a repositioned drug for the treatment of advanced PCa [259]. Even though these studies are interesting and could pave the way for other ones, we would like to highlight that the TCS role in cancer is very controversial and deserves particular attention. Indeed, in the previous paragraph we discussed research data from other studies confirming that TCS promotes several types of cancer and is involved, as well, in metastatization and resistance onset mechanisms. Our idea is that more studies are necessary in order to clarify the exact impact of TCS under an oncological point of view. Recently, topical TCS and some derivatives have been also proposed for the treatment of cutaneous leishmaniasis, an important neglected tropical infection that is caused by the protozoan *Leishmania* and affecting 12 million people in 98 countries. TCS has shown preclinical efficacy in a murine *Leishmania amazonensis* model, suggesting that the parasite plasma membrane may be a possible target of the drug, leading to an irreversible lethal antiparasitic effect [260]. Otero et al. (2017) [261] reported some triclosan-caffeic acid hybrids with antiprotozoal activity, which act by blocking the iron utilization by the parasite and the loss of mitochondrial transmembrane potential. As in the case of hypnospores of *Perkinsus* spp, TCS exhibited a high activity through the change in lipid content distributions and an increased oxidative stress [262]. Finally, the synthesis and leishmanicidal, and trypanocidal activities of a small library of triclosan–hydrazone hybrids has been reported. These compounds were evaluated against *L. panamensis* and *Trypanosoma cruzi* and showed an interesting potential as leads for the further development of drugs [263]. All together, these studies demonstrated a high potential for the use of TCS in treating intracellular parasites that are still difficult to eradicate.

## 10. Summary

TCS is a synthetic, chlorinated phenolic antimicrobial agent that is commonly used in commercial and healthcare products. As a broad-spectrum antibacterial agent, TCS has been ubiquitously added to different personal hygiene products, such as antimicrobial soaps, deodorants, mouthwashes, toothpastes, disinfectants, and cosmetics, typically at concentrations of 0.1–0.3% and also to other products such as bedding, fabrics, clothes, toys, trash bags, household cleaning products, kitchen utensils, and seafoods. Besides, there is strong evidence for TCS’s disrupting effects on the endocrine system, especially on reproductive hormones. TCS belongs to EDCs, which adversely affects the endocrine system leading to compromised functions of hormones. Epidemiological studies report a link between TCS exposure and several adverse health outcomes including alterations in thyroid function and an increased risk for hypersensitivity diseases suggesting an immunomodulatory role for the synthetic chemical. Even though TCS possesses wide biological properties that have been exploited in several applications, there are many important considerations that need attention and represent severe issues that should be taken into account and diminish its indiscriminate and widespread use. Amongst them, the ascertained rise of bacterial resistance, the high impact on gut microbiota, immune and endocrine system, different organs, with related negative effects on human health, and, ultimately, its environmental accumulation as a polluting substance represent critical aspects. On the contrary, under a scientific research point of view, it is desirable to study TCS as repositioning drug, considering some interesting preliminary studies regarding its potential anticancer and antiparasitic properties. This review provides the main information and highlights advantages and disadvantages regarding the different TCS applications, inviting to a more rationale and limited use and unveil the TCS potential as repositioning drug.

## Data Availability

Not applicable.

## Abbreviations

AChE	Acetylcholinesterase
AhR	Aryl Hydrocarbon Receptor
AMH	Anti-Müllerian hormone
BALF	Bronchoalveolar Lavage Fluid
BMI	Body Mass Index
CEC	Contaminants of Emerging Concern
CNS	Central Nervous System
2,8-DCDD	2,8-dichlorodibenzo-*p*-dioxin
2,4-DCP	2,4-Dichlorophenol
EDCs	Endocrine Disrupting Chemicals
EDTA	Ethylenediaminetetraacetic Acid
ENR	Enoyl-acyl carrier Protein Reductase
EU	European Union
FASN	Fatty Acid Synthase Inhibitor
FDA	Food and Drug Administration
FSH	Follicle-stimulating hormone
FT3	Triiodothyronine
FT4	Thyroxine
GOT	Glutamic Oxaloacetic Transaminase
GPT	Glutamic Pyruvic Transaminase
HCC	Hepatocellular Carcinoma
HOME	Health Outcomes and Measures of the Environment
HUVEC	Human Umbilical Vein Endothelial Cell
IC_50_	Half Minimal Inhibitory Concentration
IgE	Immunoglobulin E
IL-6	Interleukin-6
LU	Luteinizing hormone
LPS	Lipopolysaccharide
MIC	Minimum Inhibitory Concentration
MTCS	Methyltriclosan
NHANES	National Health and Nutrition Examination Surveys
OTC	Over-the-counter
PCOS	Polycystic Ovary Syndrome
PPCPs	Pharmaceutical and Personal Care Products
PVM/MA	Polyvinylmethyl Ether Maleic Acid
REACH	Registration, Evaluation, Authorisation and Restriction of Chemicals
ROS	Reactive Oxygen Species
SHIME	Simulator of the Human Intestinal Microbial Ecosystem
SSIs	Surgical Site Infections
TCC	Triclocarban
TCS	Triclosan
TNF-α	Tumor Necrosis Factor alpha
TP	Total Protein
TSH	Thyroid-stimulating hormone
UDP	Uridine diphosphate
UGTs	UDP-glucuronosyltransferases

## References

[1] Halden R.U., Lindeman A.E., Aiello A.E., Andrews D., Arnold W.A., Fair P., Fuoco R.E., Geer L.A., Johnson P.I., Lohmann R. (2017). The Florence statement on triclosan and triclocarban. Environ. Health Perspect..

[2] Weatherly L.M., Gosse J.A. (2017). Triclosan exposure, transformation, and human health effects. J. Toxicol. Environ. Health B Crit. Rev..

[3] Gálvez-Ontiveros Y., Páez S., Monteagudo C., Rivas A. (2020). Endocrine disruptors in food: Impact on gut microbiota and metabolic diseases. Nutrients.

[4] Catalano A., Iacopetta D., Sinicropi M.S., Franchini C. (2021). Diarylureas as antitumor agents. Appl. Sci..

[5] Catalano A. (2022). Diarylurea: A privileged scaffold in drug discovery and therapeutic development. Curr. Med. Chem..

[6] Iacopetta D., Catalano A., Ceramella J., Saturnino C., Salvagno L., Ielo I., Drommi D., Scali E., Plutino M.R., Rosace G. (2021). The different facets of triclocarban: A review. Molecules.

[7] Lee J.D., Lee J.Y., Kwack S.J., Shin C.Y., Jang H.J., Kim H.Y., Kim M.K., Seo D.W., Lee B.M., Kim K.B. (2019). Risk assessment of triclosan, a cosmetic preservative. Toxicol. Res..

[8] Wang Y., Liang W. (2021). Occurrence, toxicity, and removal methods of triclosan: A timely review. Curr. Poll. Rep..

[9] Gao C.J., Jia L.L., Guo Y. (2018). Triclosan in over the counter medicines of South China. Environ. Monit. Assess..

[10] (2019). Statista. Sales of the Leading Toothpaste Brands in the United States in 2019 (in Million U.S. Dollars). https://www.statista.com/statistics/195650/leading-us-toothpaste-brands-in-2007-and-2008-based-on-sales/.

[11] (2019). Statista. Sales Growth of the Leading Toothpaste Brands in the United States in 2019 (Change to Prior Sales Year). https://www.statista.com/statistics/195651/us-sales-growth-of-toothpaste-brands-in-2007-and-2008/.

[12] Al Habashneh R., Farasin R., Khader Y. (2017). The effect of a triclosan/copolymer/fluoride toothpaste on plaque formation, gingivitis, and dentin hypersensitivity: A single-blinded randomized clinical study. Quintessence Int..

[13] Nachu S., Ravoori S., Pachava S. (2022). Antiplaque efficacy of toothpaste–A systematic review and meta-analysis of randomized controlled trials. J. Ind. Associat. Public Health Dent..

[14] Zhu Q., Wang M., Jia J., Hu Y., Wang X., Liao C., Jiang G. (2020). Occurrence, distribution, and human exposure of several endocrine disrupting chemicals in indoor dust: A nationwide study. Environ. Sci. Technol..

[15] Zhang Y., Li T.-T., Shiu B.-C., Sun F., Ren H.-T., Zhang X., Lou C.-W., Lin J.-H. (2021). Eco-friendly versatile protective polyurethane/triclosan coated polylactic acid nonwovens for medical covers application. J. Clean. Prod..

[16] Ahmed I., Boulton A.J., Rizvi S., Carlos W., Dickenson E., Smith N.-A., Reed M. (2019). The use of triclosan-coated sutures to prevent surgical site infections: A systematic review and meta-analysis of the literature. BMJ Open.

[17] Daoud F.C., Coppry M., Moore N., Rogues A.M. (2022). Do triclosan sutures modify the microbial diversity of surgical site infections? a systematic review and meta-analysis. Microorganisms.

[18] Alfhili M.A., Lee M.H. (2019). Triclosan: An update on biochemical and molecular mechanisms. Oxid. Med. Cell. Longev..

[19] Iacopetta D., Ceramella J., Catalano A., Saturnino C., Pellegrino M., Mariconda A., Longo P., Sinicropi M.S., Aquaro S. (2022). COVID-19 at a glance: An up-to-date overview on variants, drug design and therapies. Viruses.

[20] Chu W., Fang C., Deng Y., Xu Z. (2020). Intensifed disinfection amid COVID-19 pandemic poses potential risks to water quality and safety. Environ. Sci. Technol..

[21] Kumar S., Paul T., Shukla S.P., Kumar K., Karmakar S., Bera K.K. (2021). Biomarkers-based assessment of triclosan toxicity in aquatic environment: A mechanistic review. Environ. Pollut..

[22] Tan Q., Chen J., Chu Y., Liu W., Yang L., Ma L., Zhang Y., Qui D., Wu Z., He F. (2021). Triclosan weakens the nitrification process of activated sludge and increases the risk of the spread of antibiotic resistance genes. J. Hazard. Mater..

[23] Catalano A., Iacopetta D., Ceramella J., Scumaci D., Giuzio F., Saturnino C., Aquaro S., Rosano C., Sinicropi M.S. (2022). Multidrug resistance (MDR): A widespread phenomenon in pharmacological therapies. Molecules.

[24] Usman M., Farooq M., Hanna K. (2020). Environmental side effects of the injudicious use of antimicrobials in the era of COVID-19. Sci. Total Environ..

[25] Alvarez-Munoz D., Rodriguez-Mozaz S., Jacobs S., Serra-Compte A., Caceres N., Sioen I., Verbeke W., Barbosa V., Ferrari F., Fernandez-Tejedor M. (2018). Pharmaceuticals and endocrine disruptors in raw and cooked seafood from european market: Concentrations and human exposure levels. Environ. Int..

[26] Ates G., Goldberg J., Currais A., Maher P. (2020). CMS121, a fatty acid synthase inhibitor, protects against excess lipid peroxidation and inflammation and alleviates cognitive loss in a transgenic mouse model of Alzheimer's disease. Redox Biol..

[27] (2010). European Commission, Scientific Committees on Consumer Safety (SCCS) Opinion on Triclosan, Antimicrobial Resistance. (SCCP/1251/09). Directorate-General for Health and Consumers, Opinion Approved 7th Plenary. http://ec.europa.eu/health/scientific_committees/consumer_safety/docs/sccs_o_023.pdf.

[28] Lubarsky H.V., Gerbersdorf S.U., Hubas C., Behrens S., Ricciardi F., Paterson D.M. (2012). Impairment of the bacterial biofilm stability by triclosan. PLoS ONE.

[29] Liu X., Tu M., Wang S., Wang Y., Wang J., Hou Y., Zheng X., Yan Z. (2021). Research on freshwater water quality criteria, sediment quality criteria and ecological risk assessment of triclosan in China. Sci. Total Environ..

[30] Contardo-Jara V., Meinecke S., Feibicke M., Berghahn R., Schmidt R., Mohr S. (2021). Fate, bioaccumulation and toxic effects of triclosan on a freshwater community–A mesocosm study. Environ. Adv..

[31] Mohan S., Balakrishnan P. (2019). Triclosan in treated wastewater from a city wastewater treatment plant and its environmental risk assessment. Water Air Soil Pollut..

[32] Dodson R.E., Boronow K.E., Susmann H., Udesky J.O., Rodgers K.M., Weller D., Woudneh M., Brody J.G., Rudel R.A. (2020). Consumer behavior and exposure to parabens, bisphenols, triclosan, dichlorophenols, and benzophenone-3: Results from a crowdsourced biomonitoring study. Int. J. Hygiene Environ. Health.

[33] Di Poi C., Costil K., Bouchart V., Halm-Lemeille M.P. (2018). Toxicity assessment of five emerging pollutants, alone and in binary or ternary mixtures, towards three aquatic organisms. Environ. Sci. Pollut. Res..

[34] Dar O.I., Aslam R., Pan D., Sharma S., Andotra M., Kaur A., Jia A.Q., Faggio C. (2022). Source, bioaccumulation, degradability and toxicity of triclosan in aquatic environments: A review. Environ. Technol. Innov..

[35] Lu S., Yu Y., Ren L., Zhang X., Liu G., Yu Y. (2018). Estimation of intake and uptake of bisphenols and triclosan from personal care products by dermal contact. Sci. Total Environ..

[36] Skarha J., Mínguez-Alarcón L., Williams P.L., Korevaar T.I.M., de Poortere R.A., Broeren M.A.C., Ford J.B., Eliot M., Hauser R., Braun J.M. (2019). Cross-sectional associations between urinary triclosan and serum thyroid function biomarker concentrations in women. Environ. Int..

[37] Arbuckle T.E., Marro L., Davis K., Fisher M., Ayotte P., Belanger P., Dumas P., LeBlanc A., Berube R., Gaudreau E. (2015). Exposure to free and conjugated forms of bisphenol A and triclosan among pregnant women in the MIREC cohort. Environ. Health Perspect..

[38] Binder A.M., Corvalan C., Calafat A.M., Ye X., Mericq V., Pereira A., Michels K.B. (2018). Childhood and adolescent phenol and phthalate exposure and the age of menarche in Latina girls. Environ. Health.

[39] Savage J.H., Matsui E.C., Wood R.A., Keet C.A. (2012). Urinary levels of triclosan and parabens are associated with aeroallergen and food sensitization. J. Allergy Clin. Immunol..

[40] Alfhili M.A., Hussein H.A., Park Y., Lee M.H., Akula S.M. (2021). Triclosan induces apoptosis in Burkitt lymphoma-derived BJAB cells through caspase and JNK/MAPK pathways. Apoptosis.

[41] Anderson S.E., Meade B.J., Long C.M., Lukomska E., Marshall N.B. (2016). Investigations of immunotoxicity and allergic potential induced by topical application of triclosan in mice. J. Immunotoxicol..

[42] Dinwiddie M.T., Terry P.D., Chen J. (2014). Recent evidence regarding triclosan and cancer risk. Int. J. Environ. Res. Public Health.

[43] Singh S., Karthikeyan C., Moorthy N.H.N. (2020). Recent advances in the development of fatty acid synthase inhibitors as anticancer agents. Mini Rev. Med. Chem..

[44] Turanli B., Grøtli M., Boren J., Nielsen J., Uhlen M., Arga K.Y., Mardinoglu A. (2018). Drug repositioning for effective prostate cancer treatment. Front. Physiol..

[45] Montaseri H., Forbes P.B.C. (2016). A review of monitoring methods for triclosan and its occurrence in aquatic environments. Trace Trend Anal. Chem..

[46] Woodruff T.J., Zota A.R., Schwartz J.M. (2011). Environmental chemicals in pregnant women in the United States: NHANES 2003–2004. Environ. Health Perspect..

[47] Casas L., Fernandez M.F., Llop S., Guxens M., Ballester F., Olea N., Irurzun M.B., Rodriguez L.S., Riano I., Tardon A. (2011). Urinary concentrations of phthalates and phenols in a population of Spanish pregnant women and children. Environ. Int..

[48] (2016). FDA (U.S. Food and Drug Administration). 21 CFR Part 310 safety and effectiveness of consumer antiseptics. topical antimicrobial drug products for over-the-counter human use. Final rule. Fed. Reg..

[49] (2015). ECHA (European Chemicals Agency). Biocidal Products Committee (BPC): Opinion on the Application for Approval of the Active Substance: Triclosan Product-Type: 1. https://echa.europa.eu/documents/10162/efc985e4-8802-4ebb-8245-29708747a358.

[50] (2016). EC (European Commission). Commission Implementing Decision (EU) 2016/110 of 27 January 2016 Not Approving Triclosan as an Existing Active Substance for Use in Biocidal Products for Product-Type 1. http://eur-lex.europa.eu/legal-content/EN/TXT/PDF/?uri=CELEX:32016D0110&from=EN.

[51] European Union. Official Journal L359—EUR-Lex. http://eur-lex.europa.eu/legal-content/EN/TXT/PDF/?uri=OJ:L:2014:107:FULL&from=EN.

[52] European Commission. Fitness Check on Endocrine Disruptors. Commission Staf Working Document. SWD (2020) 251 Fnal. https://ec.europa.eu/environment/pdf/chemicals/2020/10/SWD_on_Endocrines_disruptors.pdf.

[53] (2020). National Institute of Environmental Health Sciences (NIEHS). Endocrine Disruptors. https://www.niehs.nih.gov/health/topics/agents/endocrine/index.cfm.

[54] (2020). European Chemical Agency (ECHA). Substance Infocard, Triclosan. https://echa.europa.eu/substance-information/-/substanceinfo/100.020.167.

[55] Ahmed I., Lin H., Zou L., Brody A.L., Li Z., Qazi I.M., Pavase T.R., Lv L. (2017). A comprehensive review on the application of active packaging technologies to muscle foods. Food Control.

[56] Schumann B., Schmid M. (2018). Packaging concepts for fresh and processed meat Recent progresses. Inn. Food Sci. Emerg. Technol..

[57] Beiras R., Verdejo E., Campoy-López P., Vidal-Liñán L. (2021). Aquatic toxicity of chemically defined microplastics can be explained by functional additives. J. Hazard. Mater..

[58] Petersen R.C. (2016). Triclosan antimicrobial polymers. AIMS Mol. Sci..

[59] Glaser A. (2004). The ubiquitous triclosan. A common antibacterial agent exposed. Pest. You.

[60] O'Neal T.K. (2019). Identification and Characterization of Triclosan Resistant Bacteria. Ph.D. Dissertation.

[61] European Commission. Commission Decision of 19 March 2010 Concerning the Non-Inclusion of 2,4,4’-trichloro-2’-hydroxydiphenyl ether in the Union List of Additives Which May Be Used in the Manufacture of Plastic Materials and Articles Intended to Come into Contact with Foodstuffs under Directive 2002/72/EC (Notified under Document C(2010) 1613) (Text with EEA Relevance) (2010/169/EU). https://www.legislation.gov.uk/eudn/2010/169/2010-03-19.

[62] Marazuela M.D., Klaiber M., Moreno-Gordaliza E., Barata A., Gómez-Gómez M.M. (2022). Safety assessment of commercial antimicrobial food packaging: Triclosan and microplastics, a closer look. Food Pack. Shelf Life.

[63] Shrestha P., Zhang Y., Chen W.J., Wong T.Y. (2020). Triclosan: Antimicrobial mechanisms, antibiotics interactions, clinical applications, and human health. J. Environ. Sci. Health Part. C.

[64] Adkins J.M., Ahmar R.A., Yu H.D., Musick S.T., Alberico A.M. (2022). Comparison of antimicrobial activity between bacitracin-soaked sutures and triclosan coated suture. J. Surg. Res..

[65] Orhan M. (2020). Triclosan applications for biocidal functionalization of polyester and cotton surfaces. J. Eng. Fib. Fabr..

[66] Suarez S., Dodd M.C., Omil F., von Gunten U. (2007). Kinetics of triclosan oxidation by aqueous ozone and consequent loss of antibacterial activity: Relevance to municipal wastewater ozonation. Water Res..

[67] Querido M.M., Rosário F., Bessa M.J., Mendes F., Teixeira J.C., Teixeira J.P., Pereira C.C. (2022). In vitro cyto- and genotoxicity assessment of antibacterial paints with triclosan and isoborneol. Toxics.

[68] Schweizer H.P. (2001). Triclosan: A widely used biocide and its link to antibiotics. FEMS Microb. Lett..

[69] Karnas K., Marotta J., Koseki R., Sherman E., Exton L.P. (2019). Triclosan resistance derived across environmentally and clinically relevant Gram negative bacteria. J. Pennsylv. Acad. Sci..

[70] Halden R.U. (2014). On the need and speed of regulating triclosan and triclocarban in the United States. Environ. Sci. Technol..

[71] Møretrø T., Høiby-Pettersen G.S., Habimana O., Heir E., Langsrud S. (2011). Assessment of the antibacterial activity of a triclosan-containing cutting board. Int. J. Food. Microb..

[72] Gowda J., Tavarageri A., Kulkarni R., Anegundi R.T., Janardhan A., Bhat M.A. (2021). Comparative assessment of the antimicrobial efficacy of triclosan, amoxicillin and eugenol against *Enterococcus faecalis*. Int. J. Clin. Ped. Dent..

[73] Pozzi C., Ferrari S., Cortesi D., Luciani R., Stroud R.M., Catalano A., Costi M.P., Mangani S. (2012). The structure of *Enterococcus faecalis* thymidylate synthase provides clues about folate bacterial metabolism. Acta Crystallogr. D Biol. Crystallogr..

[74] Zeng W., Xu W., Xu Y., Liao W., Zhao Y., Zheng X., Xu C., Zhou T., Cao J. (2020). The prevalence and mechanism of triclosan resistance in *Escherichia coli* isolated from urine samples in Wenzhou, China. Antimicrob. Resist. Infect. Control..

[75] Rozman U., Pušnik M., Kmetec S., Duh D., Šostar Turk S. (2021). Reduced Susceptibility and Increased Resistance of Bacteria against Disinfectants: A Systematic Review. Microorganisms.

[76] Franklyne J.S., Ebenazer A., Mukherjee A., Chandrasekaran N. (2021). Role of triclosan microemulsion against triclosan resistant clones of bacterial pathogens. J. Drug Deliv. Sci. Technol..

[77] Araujo C.B., Ribeiro A.B., Fortes C.V., Bueno F.L., De Wever B., Oliveira V.C., Macedo A.P., Paranhos H.F.O., Lovato da Silva C.H. (2021). Effect of local hygiene protocols on denture-related stomatitis, biofilm, microbial load, and odor: A randomized controlled trial. J. Prosthet. Dent..

[78] Jongsma M.A., Van Der Mei H.C., Atema-Smit J., Busscher H.J., Ren Y. (2015). In vivo biofilm formation on stainless steel bonded retainers during different oral health-care regimens. Int. J. Oral Sci..

[79] Maiden M.M., Hunt A.M.A., Zachos M.P., Gibson J.A., Hurwitz M.E., Mulks M.H., Waters C.M. (2018). Triclosan is an aminoglycoside adjuvant for eradication of *Pseudomonas aeruginosa* biofilms. Antimicrob. Agents Chemother..

[80] Maiden M.M., Waters C.M. (2020). Triclosan depletes the membrane potential in *Pseudomonas aeruginosa* biofilms inhibiting aminoglycoside induced adaptive resistance. PLoS Pathog..

[81] Ayyash M., Shehabi A.A., Mahmoud N.N., Al-Bakri A.G. (2019). Antibiofilm properties of triclosan with EDTA or cranberry as Foley Catheter lock solutions. J. Appl. Microbiol..

[82] Talaat D.M., Sharaf A.A.E.A., Ghoneim M.A.E.M., El-Shazly S.A., El Meligy O.A.E.S. (2018). Efficacy of two mouth rinse sprays in inhibiting Streptococcus mutans growth on toothbrush bristles. Saudi Dent. J..

[83] Yadav S., Mandal H., Saravanan V., Das P., Singh S.K. (2021). In vitro and in silico analysis of *L. donovani* enoyl acyl carrier protein reductase-A possible drug target. J. Biomol. Srtuct. Dynam..

[84] Vosatka R., Kratky M., Vinsova J. (2018). Triclosan and its derivatives as antimycobacterial active agents. Eur. J. Pharm. Sci..

[85] Bilsland E., van Vliet L., Williams K., Feltham J., Carrasco M.P., Fotoran W.L., Cubillos E.F.G., Wunderlich G., Grøtli M., Hollfelder F. (2018). Plasmodium dihydrofolate reductase is a second enzyme target for the antimalarial action of triclosan. Sci. Rep..

[86] Chetty S., Armstrong T., Sharma Kharkwal S., Drewe W.C., De Matteis C.I., Evangelopoulos D., Bhakta S., Thomas N.R. (2021). New InhA inhibitors based on expanded triclosan and di-triclosan analogues to develop a new treatment for tuberculosis. Pharmaceuticals.

[87] de Luco J.F., Recio-Balsells A.I., Ghiano D.G., Bortolotti A., Belardinelli J.M., Liu N., Hoffmann P., Lherbet C., Tonge P.J., Tekwani W. (2021). Exploring the chemical space of 1,2,3-triazolyl triclosan analogs for discovery of new antileishmanial chemotherapeutic agents. RSC Med. Chem..

[88] Walsh L.J., Healey D.L. (2019). Prevention and caries risk management in teenage and orthodontic patients. Austr. Dent. J..

[89] Hall P.J., Green A.K., Horay C.P., de Brabander S., Beasley T.J., Cromwell V.J., Holt J.S., Savage D.J. (2003). Plaque antibacterial levels following controlled food intake and use of a toothpaste containing 2% zinc citrate and 0.3% Triclosan. Int. Dent. J..

[90] Riley P., Lamont T. (2013). Triclosan/copolymer containing toothpastes for oral health (Review). Cochrane Database Syst. Rev..

[91] Singh S., Chaknis P., DeVizio W., Petrone M., Panagakos F.S., Proskin H.M. (2010). A Clinical investigation of the efficacy of three commercially available dentifrices for controlling established gingivitis and supragingival plaque. J. Clin. Dent..

[92] West N.X., He T., Hellin N., Claydon N., Seong J., Macdonald E., Farrell S., Eusebio R., Wilberg A. (2019). Randomized in situ clinical trial evaluating erosion protection efficacy of a 0.454% stannous fluoride dentifrice. Int. J. Dent. Hyg..

[93] Stewart B., Shibli J.A., Araujo M., Figueiredo L.C., Panagakos F., Matarazzo F., Mairink R., Onuma T., Faveri M., Retamal-Valdes B. (2020). Effects of a toothpaste containing 0.3% triclosan on periodontal parameters of subjects enrolled in a regular maintenance program: A secondary analysis of a 2-year randomized clinical trial. J. Periodontol..

[94] Fine D.H., Sreenivasan P.K., McKiernan M., Tischio-Bereski D., Furgang D. (2012). Whole mouth antimicrobial effects after oral hygiene: Comparison of three dentifrice formulations. J. Clin. Periodont..

[95] Panagakos F.S., Volpe A.R., Petrone M.E., DeVizio W., Davies R.M., Proskin H.M. (2005). Advanced oral antibacterial/anti-inflammatory technology: A comprehensive review of the clinical benefits of a triclosan/copolymer/fluoride dentifrice. J. Clin. Dent..

[96] Kerdvongbundit V., Wikesjö U.M. (2003). Effect of triclosan on healing following non-surgical periodontal therapy in smokers. J. Clin. Periodontol..

[97] Aminu N., Yam M.F., Chan S.Y., Bello I., Umar N.M., Nuhu T., Toh S.M. (2021). The evaluation of healing effect of triclosan and flurbiprofen-loaded nanogels in experimental periodontitis in rats by morphometric analysis. Saudi Dent. J..

[98] Shu W., Zhang Y., Zhang C., You Q., Zhou H., Wen S. (2021). Triclosan inhibits the activation of human periodontal ligament fibroblasts induced by lipopolysaccharide from *Porphyromonas gingivalis*. J. Biomed. Res..

[99] Ceramella J., Iacopetta D., Catalano A., Cirillo F., Lappano R., Sinicropi M.S. (2022). A review on the antimicrobial activity of Schiff bases: Data collection and recent studies. Antibiotics.

[100] Pavez L., Tobar N., Chacon C., Arancibia R., Martínez C., Tapia C., Pastor A., Gonzàlez M., Martínez J., Smith P.C. (2018). Chitosan-triclosan particles modulate inflammatory signaling in gingival fibroblasts. J. Periodont. Res..

[101] Li X., Zhong Y., He W., Huang S., Li Q., Guo C., Ma S., Li G., Yu Y. (2021). Co-exposure and health risks of parabens, bisphenols, triclosan, phthalate metabolites and hydroxyl polycyclic aromatic hydrocarbons based on simultaneous detection in urine samples from Guangzhou, South China. Environ. Pollut..

[102] Milanović M., Đurić L., Milošević N., Milić N. (2021). Comprehensive insight into triclosan—from widespread occurrence to health outcomes. Environ. Sci. Pollut. Res..

[103] Allmyr M., Panagiotidis G., Sparve E., Diczfalusy U., Sandborgh-Englund G. (2009). Human exposure to triclosan via toothpaste does not change CYP3A4 activity or plasma concentrations of thyroid hormones. Basic Clin. Pharmacol. Toxicol..

[104] Sandborgh-Englund G., Adolfsson-Erici M., Odham G., Ekstrand J. (2006). Pharmacokinetics of triclosan following oral ingestion in humans. J. Toxicol. Environ. Health A.

[105] Queckenberg C., Meins J., Wachall B., Doroshyenko O., Tomalik-Scharte D., Bastian B., Abdel-Tawab M., Fuhr U. (2010). Absorption, pharmacokinetics, and safety of triclosan after dermal administration. Antimicrob. Agents Chemother..

[106] Dhillon G.S., Kaur S., Pulicharla R., Brar S.K., Cledón M., Verma M., Surampalli R.Y. (2015). TCS: Current status, occurrence, environmental risks and bioaccumulation potential. Int. J. Environ. Res. Publ. Health.

[107] Scientific Committee on Consumer Products (SCCP). Scientific Committee on Consumer Safety. Opinion on Triclosan Antimicrobial Resistance; SCCP/1251/09. https://ec.europa.eu/health/sites/health/files/scientific_committees/consumer_safety/docs/sccs_o_023.pdf.

[108] Bester K. (2005). Fate of triclosan and triclosan-methyl in sewage treatment plants and surface waters. Arch. Environ. Contam. Toxicol..

[109] (2009). Scientific Committee on Consumer Products (SCCP). Opinion on Triclosan (COLIPA No. P32). https://ec.europa.eu/health/ph_risk/committees/04_sccp/docs/sccp_o_166.pdf.

[110] Dix-Cooper L., Kosatsky T. (2019). Use of antibacterial toothpaste is associated with higher urinary triclosan concentrations in Asian immigrant women living in Vancouver, Canada. Sci. Total Environ..

[111] DeLorenzo M.E., Keller J.M., Arthur C.D., Finnegan M.C., Harper H.E., Winder V.L., Zdankiewicz D.L. (2008). Toxicity of the antimicrobial compound triclosan and formation of the metabolite methyl-triclosan in estuarine systems. Environ. Toxicol..

[112] Armstrong D.L., Lozano N., Rice C.P., Ramirez M., Torrents A. (2018). Degradation of triclosan and triclocarban and formation of transformation products in activated sludge using benchtop bioreactors. Environ. Res..

[113] Chen X., Casas M.E., Nielsen J.L., Wimmer R., Bester K. (2015). Identification of Triclosan-*O*-Sulfate and other transformation products of Triclosan formed by activated sludge. Sci. Total Environ..

[114] Lozano N., Rice C.P., Ramirez M., Torrents A. (2012). Fate of Triclosan and Methyltriclosan in soil from biosolids application. Environ. Pollut..

[115] Fu J., Tan Y.X.R., Gong Z., Bae S. (2020). The toxic effect of triclosan and methyl-triclosan on biological pathways revealed by metabolomics and gene expression in zebrafish embryos. Ecotoxicol. Environ. Saf..

[116] Chen H.C., Chang J.W., Sun Y.C., Chang W.T., Huang P.C. (2022). Determination of parabens, bisphenol a and its analogs, triclosan, and benzophenone-3 levels in human urine by isotope-dilution-UPLC-MS/MS method followed by supported liquid extraction. Toxics.

[117] Fang J.L., Stingley R.L., Beland F.A., Harrouk W., Lumpkins D.L., Howard P. (2010). Occurrence, efficacy, metabolism, and toxicity of triclosan. J. Environ. Sci. Health Part. C.

[118] Zhang H., Sanidad K.Z., Zhu L., Parsonnet J., Haggerty T.D., Zhang G., Cai Z. (2021). Frequent occurrence of triclosan hydroxylation in mammals: A combined theoretical and experimental investigation. J. Hazard. Mater..

[119] Latch D.E., Packer J.L., Arnold W.A., McNeill K. (2003). Photochemical conversion of triclosan to 2, 8-dichlorodibenzo-*p*-dioxin in aqueous solution. J. Photochem. Photobiol. A Chem..

[120] Ding J., Su M., Wu C., Lin K. (2015). Transformation of triclosan to 2,8-dichlorodibenzo-*p*-dioxin by iron and manganese oxides under near dry conditions. Chemosphere.

[121] Buth J.M., Steen P.O., Sueper C., Blumentritt D., Vikesland P.J., Arnold W.A., McNeill K. (2010). Dioxin photoproducts of triclosan and its chlorinated derivatives in sediment cores. Environ. Sci. Technol..

[122] Rodrigues F., Lehmann M., doAmaral V.S., Reguly M.L., de Andrade H.H.R. (2007). Genotoxicity of three mouthwash products, Cepacol^®^, Periogard^®^, and Plax^®^, in the Drosophila wing-spot test. Environ. Mol. Mutagen..

[123] Carrisi C., Madeo M., Morciano P., Dolce V., Cenci G., Cappello A.R., Mazzeo G., Iacopetta D., Capobianco L. (2008). Identification of the *Drosophila melanogaster* mitochondrial citrate carrier: Bacterial expression, reconstitution, functional characterization and developmental distribution. J. Biochem..

[124] (2017). European Chemicals Agency (ECHA). Guidance on Information Requirements and Chemical Safety Assessment, Part C: PBT/vPvB assessment, Version 3.0. https://echa.europa.eu/information-on-chemicals/euclef.

[125] Li L. (2021). Toxicity evaluation and by-products identifcation of triclosan ozonation and chlorination. Chemosphere.

[126] Weiss L., Arbuckle T.E., Fisher M., Ramsay T., Mallick R., Hauser R., LeBlanc A., Walker M., Dumas P., Lang C. (2015). Temporal variability and sources of triclosan exposure in pregnancy. Int. J. Hygiene Environ. Health.

[127] Johnson P.I., Koustas E., Vesterinen H.M., Sutton P., Atchley D.S., Kim A.N., Campbell M., Donald J.M., Sen S., Bero L. (2016). Application of the Navigation Guide systematic review methodology to the evidence for developmental and reproductive toxicity of triclosan. Environ. Int..

[128] Paul K.B., Thompson J.T., Simmons S.O., Vanden Heuvel J.P., Crofton K.M. (2013). Evidence for triclosan-induced activation of human and rodent xenobiotic nuclear receptors. Toxicol. In Vitro.

[129] Koeppe E.S., Ferguson K.K., Colacino J.A., Meeker J.D. (2013). Relationship between urinary triclosan and paraben concentrations and serum thyroid measures in NHANES 2007–2008. Sci. Total Environ..

[130] Cullinan M.P., Palmer J.E., Carle A.D., West M.J., Seymour G.J. (2012). Long term use of triclosan toothpaste and thyroid function. Sci. Total Environ..

[131] Taha M., Marie A.M., Ahmed-Farid O.A. (2020). Combined approaches for evaluation of xenoestrogen neural toxicity and thyroid dysfunction: Screening of oxido-nitrosative markers, DNA fragmentation, and biogenic amine degradation. J. Biochem. Mol. Toxicol..

[132] Zhang P., Yang M., Zeng L., Liu C. (2018). P38/TRHr-dependent regulation of TPO in thyroid cells contributes to the hypothyroidism of triclosan-treated rats. Cell. Physiol. Biochem..

[133] Cao X.Y., Hua X., Xiong J.W., Zhu W.T., Zhang J., Chen L. (2018). Impact of triclosan on female reproduction through reducing thyroid hormones to suppress hypothalamic kisspeptin neurons in mice. Front. Mol. Neurosci..

[134] Abd-Elhakim Y.M., Mohammed A.T., Ali H.A. (2018). Impact of subchronic exposure to triclosan and/or fuoride on estrogenic activity in immature female rats: The expression pattern of calbindin-D9k and estrogen receptor α genes. J. Biochem. Mol. Toxicol..

[135] Jurewicz J., Wielgomas B., Radwan M., Karwacka A., Klimowska A., Dziewirska E., Korczak K., Zajdel R., Radwan P., Hanke W. (2019). Triclosan exposure and ovarian reserve. Reprod. Toxicol..

[136] Chen W., Yang X., Wang B., Wang L., Yu X. (2019). The effects and possible mechanisms of triclosan on steroidogenesis in primary rat granulosa cells. Reprod. Toxicol..

[137] Du Y., Wang B., Cai Z., Zhang H., Wang B., Liang W., Zhou G., Ouyang F., Wang W. (2021). The triclosan-induced shift from aerobic to anaerobic metabolism link to increased steroidogenesis in human ovarian granulosa cells. Ecotoxicol. Environ. Safety.

[138] Basini G., Bussolati S., Bertini S., Quintavalla F., Grasselli F. (2021). Evaluation of triclosan effects on cultured swine luteal cells. Animals.

[139] Rehman S., Usman Z., Rehman S., Aldraihem M., Rehman N., Rehman I., Ahmad G. (2018). Endocrine disrupting chemicals and impact on male reproductive health. Transl. Androl. Urol..

[140] Gee R.H., Charles A., Taylor N., Darbre P.D. (2008). Oestrogenic and androgenic activity of triclosan in breast cancer cells. J. Appl. Toxicol..

[141] Yawer A., Sychrová E., Labohá P., Raška J., Jambor T., Babica P., Sovadinová I. (2020). Endocrine-disrupting chemicals rapidly affect intercellular signaling in Leydig cells. Toxicol. Appl. Pharmacol..

[142] Kumar V., Chakraborty A., Kural M.R., Roy P. (2009). Alteration of testicular steroidogenesis and histopathology of reproductive system in male rats treated with triclosan. Reprod. Toxicol..

[143] Lan Z., Hyung Kim T., Shun Bi K., Hui Chen X., Sik Kim H. (2015). Triclosan exhibits a tendency to accumulate in the epididymis and shows sperm toxicity in male sprague-dawley rats. Environ. Toxicol..

[144] Priyanka T.A., Maske P., Mote C., Dighe V. (2020). Gestational and lactational exposure to triclosan causes impaired fertility of F1 male offspring and developmental defects in F2 generation. Environ. Pollut..

[145] Ha M., Zhang P., Li L., Liu C. (2018). Triclosan suppresses testicular steroidogenesis via the miR-6321/JNK/Nur77 cascade. Cell Physiol. Biochem..

[146] Duan P., Huang X., Ha M., Li L., Liu C. (2020). miR-142-5p/DAX1-dependent regulation of P450c17 contributes to triclosan-mediated testosterone suppression. Sci. Total Environ..

[147] Zhu W., Zhang H., Tong C., Xie C., Fan G., Zhao S., Yu X., Tian Y., Zhang J. (2016). Environmental exposure to triclosan and semen quality. Int. J. Environ. Res. Public Health.

[148] Jurewicz J., Radwan M., Wielgomas B., Kałużny P., Klimowska A., Radwan P., Hanke W. (2018). Environmental levels of triclosan and male fertility. Environ. Sci. Pollut. Res. Int..

[149] Nassan F.L., Mínguez-Alarcón L., Williams P.L., Dadd R., Petrozza J.C., Ford J.B., Calafat A.M., Hauser R. (2019). Urinary triclosan concentrations and semen quality among men from a fertility clinic. Environ. Res..

[150] Pernoncini K.V., Montagnini B.G., de Góes M.L.M., Garcia P.C., Gerardin D.C.C. (2018). Evaluation of reproductive toxicity in rats treated with triclosan. Reprod. Toxicol..

[151] Yan J., Joseph M.A., Reynolds S.A., Geer L.A. (2020). Association between urinary triclosan and serum testosterone levels in U.S. adult males from NHANES, 2011–2012. Int. J. Environ. Res. Public Health.

[152] Yuan G., Ma Y., Zeng Y., Pan H., Liu P., Liu Y., Liu G., Cheng J., Guo Y. (2022). Associations between low-dose triclosan exposure and semen quality in a Chinese population. Environ. Pollut..

[153] Scinicariello F., Buser M.C. (2016). Serum testosterone concentrations and urinary bisphenol A, benzophenon-3, triclosan, and paraben levels in male and female children and adolescents: NHANES 2011–2012. Environ. Health Perspect..

[154] Gishti O., Jaddoe V.W.V., Duijts L., Steegers E., Reiss I., Hofman A., Wong T.Y., Ikram M.K., Gaillard R. (2015). Impact of birth parameters and early life growth patterns on retinal microvascular structure in children: The Generation R Study. J. Hypertens..

[155] Toemen L., de Jonge L.L., Gishti O., van Osch-Gevers L., Taal H.R., Steegers E.A.P., Hofman A., Helbing W.A., Jaddoe V.W.V. (2016). Longitudinal growth during fetal life and infancy and cardiovascular outcomes at school-age. J. Hypertens..

[156] Wang X., Chen X., Feng X., Chang F., Chen M., Xia Y., Chen L. (2015). Triclosan causes spontaneous abortion accompanied by decline of estrogen sulfotransferase activity in humans and mice. Sci. Rep..

[157] Ouyang F., Tang W.N., Zhang H., Wang X., Zhao S., Wang W., Zhang J., Cheng W. (2018). Maternal urinary triclosan level, gestational diabetes mellitus and birth weight in Chinese women. Sci. Total Environ..

[158] Velez M.P., Arbuckle T.E., Fraser W.D. (2015). Female exposure to phenols and phthalates and time to pregnancy: The Maternal-Infant Research on Environmental Chemicals (MIREC) Study. Fertil. Steril..

[159] Wang C., Chen L., Zhao S., Hua Y., Zhou Y., Gao Y., Wang W., Zhang J., Tian Y. (2018). Impacts of prenatal triclosan exposure on fetal reproductive hormones and its potential mechanism. Environ. Int..

[160] Zhong Q., Peng M., He J., Yang W., Huang F. (2020). Association of prenatal exposure to phenols and parabens with birth size: A systematic review and meta-analysis. Sci. Total Environ..

[161] Khoshhali M., Amin M., Fatehizadeh A., Ebrahimi A., Taheri E., Kelishadi R. (2020). Impact of prenatal triclosan exposure on gestational age and anthropometric measures at birth: A systematic review and meta-analysis. J. Res. Med. Sci..

[162] Patti M.A., Henderson N.B., Gajjar P., Eliot M., Jackson-Browne M., Braun J.M. (2021). Gestational triclosan exposure and infant birth weight: A systematic review and meta-analysis. Environ. Intern..

[163] Lassen T.H., Frederiksen H., Kyhl H.B., Swan S.H., Main K.M., Andersson A.M., Lind D.V., Husby S., Wohlfahrt-Veje C., Skakkebæk N.E. (2016). Prenatal triclosan exposure and anthropometric measures including anogenital distance in danish infants. Environ. Health Perspect..

[164] Geer L.A., Pycke B.F.G., Waxenbaum J., Sherer D.M., Abulafia O., Halden R.U. (2017). Association of birth outcomes with fetal exposure to parabens, triclosan and triclocarban in an immigrant population in Brooklyn, New York. J. Hazard. Mater..

[165] Huo W., Xia W., Wu C., Zhu Y., Zhang B., Wan Y., Zhou A., Qian Z., Chen Z., Jiang Y. (2018). Urinary level of triclosan in a population of Chinese pregnant women and its association with birth outcomes. Environ. Pollut..

[166] Lester F., Arbuckle T.E., Peng Y., McIsaac M.A. (2018). Impact of exposure to phenols during early pregnancy on birth weight in two Canadian cohort studies subject to measurement errors. Environ. Int..

[167] Tran D.N., Jung E.M., Yoo Y.M., Lee J.H., Jeung E.B. (2020). Perinatal exposure to triclosan results in abnormal brain development and behavior in mice. Int. J. Mol. Sci..

[168] Chen D., Liu J., Yan W., Fang K., Xia Y., Lv W., & Shi Z. (2021). Associations of prenatal exposure to triclosan and maternal thyroid hormone levels: A systematic review and meta-analysis. Front. Endocrinol..

[169] Aker A.M., Johns L., McElrath T.F., Cantonwine D.E., Mukherjee B., Meeker J.D. (2018). Associations between maternal phenol and paraben urinary biomarkers and maternal hormones during pregnancy: A repeated measures study. Environ. Int..

[170] Wang X., Ouyang F., Feng L., Wang X., Liu Z., Zhang J. (2017). Maternal urinary triclosan concentration in relation to maternal and neonatal thyroid hormone levels: A prospective study. Environ. Health Perspect..

[171] Berger K., Gunier R.B., Chevrier J., Calafat A.M., Ye X., Eskenazi B., Harley K.G. (2018). Associations of maternal exposure to triclosan, parabens, and other phenols with prenatal maternal and neonatal thyroid hormone levels. Environ. Res..

[172] Braun J.M., Chen A., Hoofnagle A., Papandonatos G.D., Jackson-Browne M., Hauser R., Romano M.E., Karagas M.R., Yolton K., Zoeller R.T. (2018). Associations of early life urinary triclosan concentrations with maternal, neonatal, and child thyroid hormone levels. Horm. Behav..

[173] Ley C., Pischel L., Parsonnet J. (2017). Triclosan and triclocarban exposure and thyroid function during pregnancy-A randomized intervention. Reprod. Toxicol..

[174] Li J., Quan X., Zhang Y., Yu T., Lei S., Huang Z., Wang Q., Song W., Yang X., Xu P. (2022). PPARγ regulates triclosan induced placental dysfunction. Cells.

[175] Derakhshan A., Shu H., Peeters R.P., Kortenkamp A., Lindh C.H., Demeneix B., Bornehag C.G., Korevaar T.I. (2019). Association of urinary bisphenols and triclosan with thyroid function during early pregnancy. Environ. Int..

[176] Jackson-Browne M.S., Papandonatos G.D., Chen A., Calafat A.M., Yolton K., Lanphear B.P., Braun J.M. (2018). Identifying vulnerable periods of neurotoxicity to triclosan exposure in children. Environ. Health Perspect..

[177] Jackson-Browne M.S., Papandonatos G.D., Chen A., Yolton K., Lanphear B.P., Braun J.M. (2019). Early-life triclosan exposure and parent-reported behavior problems in 8-year-old children. Environ. Int..

[178] Mandal T.K., Parvin N., Joo S.W., Roy P. (2020). Risk assessment of cosmetics using triclosan on future generation’s germ cell maturation via lactating mother rats. Int. J. Environ. Res. Public Health.

[179] Ruszkiewicz J.A., Li S., Rodriguez M.B., Aschner M. (2017). Is triclosan a neurotoxic agent?. J. Toxicol. Environ. Health B.

[180] Szychowski K.A., Wnuk A., Kajta M., Wójtowicz A.K. (2016). Triclosan activates aryl hydrocarbon receptor (AhR)-dependent apoptosis and affects Cyp1a1 and Cyp1b1 expression in mouse neocortical neurons. Environ. Res..

[181] Park B.K., Gonzales E.L., Yang S.M., Bang M., Choi C.S., Shin C.Y. (2016). Effects of triclosan on neural stem cell viability and survival. Biomol. Ther..

[182] Zhu L., Shao Y., Xiao H., Santiago-Schübel B., Meyer-Alert H., Schiwy S., Yin D., Hollert H., Küppers S. (2018). Electrochemical simulation of triclosan metabolism and toxicological evaluation. Sci. Total Environ..

[183] Falisse E., Voisin A.S., Silvestre F. (2017). Impacts of triclosan exposure on zebrafish early-life stage: Toxicity and acclimation mechanisms. Aquat. Toxicol..

[184] Ho J.C., Hsiao C.D., Kawakami K., William K.F. (2016). Triclosan (TCS) exposure impairs lipid metabolism in zebrafish embryos. Aquat. Toxicol..

[185] Kim J., Oh H., Ryu B., Kim U., Lee J.M., Jung C.R., Kim C., Park J.H. (2018). Triclosan affects axon formation in the neural development stages of zebrafish embryos (*Danio rerio*). Environ. Pollut..

[186] Pullaguri N., Nema S., Bhargava Y., Bhargava A. (2020). Triclosan alters adult zebrafish behavior and targets acetylcholinesterase activity and expression. Environ. Toxicol. Pharmacol..

[187] Pullaguri N., Grover P., Abhishek S., Rajakumara E., Bhargava Y., Bhargava A. (2021). Triclosan affects motor function in zebrafish larva by inhibiting *ache* and *syn2a* genes. Chemosphere.

[188] Hao Y., Meng L., Zhang Y., Chen A., Zhao Y., Lian K., Guo X., Wang X., Du Y., Wang X. (2022). Effects of chronic triclosan exposure on social behaviors in adult mice. J. Hazard. Mater..

[189] Haggard D.E., Noyes P.D., Waters K.M., Tanguay R.L. (2016). Phenotypically anchored transcriptome profiling of developmental exposure to the antimicrobial agent, triclosan, reveals hepatotoxicity in embryonic zebrafish. Toxicol. Appl. Pharmacol..

[190] Huang W., Cao G., Deng C., Chen Y., Wang T., Chen D., Cai Z. (2023). Adverse effects of triclosan on kidney in mice: Implication of lipid metabolism disorders. J. Environ. Sci..

[191] Kalloo G., Calafat A.M., Chen A., Yolton K., Lanphear B.P., Braun J.M. (2018). Early life triclosan exposure and child adiposity at 8 years of age: A prospective cohort study. Environ. Health.

[192] Xie X., Lu C., Wu M., Liang J., Ying Y., Liu K., Huang X., Zheng S., Du X., Liu D. (2020). Association between triclocarban and triclosan exposures and the risks of type 2 diabetes mellitus and impaired glucose tolerance in the National Health and Nutrition Examination Survey (NHANES 2013–2014). Environ. Int..

[193] Lankester J., Patel C., Cullen M.R., Ley C., Parsonnet J. (2013). Urinary triclosan is associated with elevated body mass index in NHANES. PLoS ONE.

[194] Cai S., Zhu J., Sun L., Fan C., Zhong Y., Shen Q., Li Y. (2019). Association between urinary triclosan with bone mass density and osteoporosis in US adult women, 2005–2010. J. Clin. Endocrinol. Metab..

[195] Han M., Wang Y., Tang C., Fang H., Yang D., Wu J., Wang H., Chen Y., Jiang Q. (2021). Association of triclosan and triclocarban in urine with obesity risk in Chinese school children. Environ. Int..

[196] Nasab H., Mirzaee M., Ebrahimpour K., Hashemi M. (2021). Association of urinary triclosan and methyl-triclosan levels with predictive indicators of cardiovascular disease and obesity in children and adolescents in 2020 (case study: Kerman, Iran). Environ. Health Eng. Manag. J..

[197] Ye J., Zhu W., Liu H., Mao Y., Jin F., Zhang J. (2018). Environmental exposure to triclosan and polycystic ovary syndrome: A cross-sectional study in China. BMJ Open.

[198] Goodman M., Naiman D.Q., Lakind J.S. (2018). Systematic review of the literature on triclosan and health outcomes in humans. Crit. Rev. Toxicol..

[199] Bertelsen R.J., Longnecker M.P., Løvik M., Calafat A.M., Carlsen K.H., London S.J., Carlsen K.C.L. (2013). Triclosan exposure and allergic sensitization in Norwegian children. Allergy.

[200] Baur R., Gandhi J., Marshall N.B., Lukomska E., Weatherly L.M., Shane H.L., Hu G., Anderson S.E. (2021). Dermal exposure to the immunomodulatory antimicrobial chemical triclosan alters the skin barrier integrity and microbiome in mice. Toxicol. Sci..

[201] Bera K.K., Kumar S., Paul T., Prasad K.P., Shukla S.P., Kumar K. (2020). Triclosan induces immunosuppression and reduces survivability of striped catfish *Pangasianodon hypophthalmus* during the challenge to a fish pathogenic bacterium *Edwardsiella tarda*. Environ. Res..

[202] Tobar S., Tordesillas L., Berin M.C. (2016). Triclosan promotes epicutaneous sensitization to peanut in mice. Clin. Transl. Allergy.

[203] Lin M.H., Chiu S.Y., Ho W.C., Chi K.H., Liu T.Y., Wang I.J. (2022). Effect of triclosan on the pathogenesis of allergic diseases among children. J. Exp. Sci. Environ. Epidemiol..

[204] Olaniyan L.W.B., Mkwetshana N., Okoh A.I. (2016). Triclosan in water, implications for human and environmental health. SpringerPlus.

[205] Di Maio A.C., Basile G., Iacopetta D., Catalano A., Ceramella J., Cafaro D., Saturnino C., Sinicropi M.S. (2022). The significant role of nutraceutical compounds in ulcerative colitis treatment. Curr. Med. Chem..

[206] Shim J.O. (2019). Recent advance in very early onset inflammatory bowel disease. Pediatr. Gastroenterol. Hepatol. Nutr..

[207] Sanidad K.Z., Xiao H., Zhang G. (2019). Triclosan, a common antimicrobial ingredient, on gut microbiota and gut health. Gut Microb..

[208] Narrowe A.B., Albuthi-Lantz M., Smith E.P., Bower K.J., Roane T.M., Vajda A.M., Miller C.S. (2015). Perturbation and restoration of the fathead minnow gut microbiome after low-level triclosan exposure. Microbiome.

[209] Gaulke C.A., Barton C.L., Proffitt S., Tanguay R.L., Sharpton T.J. (2016). Triclosan exposure is associated with rapid restructuring of the microbiome in adult zebrafish. PLoS ONE.

[210] Hu J., Raikhel V., Gopalakrishnan K., Fernandez-Hernandez H., Lambertini L., Manservisi F., Falcioni L., Bua L., Belpoggi F., Teitelbaum S.L. (2016). Effect of postnatal low-dose exposure to environmental chemicals on the gut microbiome in a rodent model. Microbiome.

[211] Gao B., Tu P., Bian X., Chi L., Ru H., Lu K. (2017). Profound perturbation induced by triclosan exposure in mouse gut microbiome: A less resilient microbial community with elevated antibiotic and metal resistomes. BMC Pharmacol. Toxicol..

[212] Ma Y., Guo Y., Ye H., Zhang J., Ke Y. (2020). Perinatal triclosan exposure in the rat induces long-term disturbances in metabolism and gut microbiota in adulthood and old age. Environ. Res..

[213] Bever C.S., Rand A.A., Nording M., Taft D., Kalanetra K.M., Mills D.A., Breck M.A., Smilowitz J.T., German J.B., Hammock B.D. (2018). Effects of triclosan in breast milk on the infant fecal microbiome. Chemosphere.

[214] Ribado J.V., Ley C., Haggerty T.D., Tkachenko E., Bhatt A.S., Parsonnet J. (2017). Household triclosan and triclocarban effects on the infant and maternal microbiome. EMBO Mol. Med..

[215] Mahalak K.K., Firrman J., Lee J.J., Bittinger K., Nuñez A., Mattei L.M., Zhang H., Fett B., Bobokalonov J., Arango-Argoty G. (2020). Triclosan has a robust, yet reversible impact on human gut microbial composition in vitro. PLoS ONE.

[216] Yang H., Wang W., Romano K.A., Gu M., Sanidad K.Z., Kim D., Yang J., Schmidt B., Panigrahy D., Pei R. (2018). A common antimicrobial additive increases colonic inflammation and colitis-associated colon tumorigenesis in mice. Sci. Translat. Med..

[217] Zhang P., Zheng L., Duan Y., Gao Y., Gao H., Mao D., Luo Y. (2022). Gut microbiota exaggerates triclosan-induced liver injury via gut-liver axis. J. Hazard. Mater..

[218] Zang L., Ma Y., Huang W., Ling Y., Sun L., Wang X., Zeng A., Dahlgren R.A., Wang C., Wang H. (2019). Dietary *Lactobacillus plantarum* ST-III alleviates the toxic effects of triclosan on zebrafish (*Danio rerio*) via gut microbiota modulation. Fish. Shellfish Immunol..

[219] Maksymowicz M., Machowiec P.A., Ręka G., Korzeniowska A., Leszczyk P., Piecewicz Szczęsna H. (2021). Mechanism of action of triclosan as an endocrine-disrupting chemical with its impact on human health–literature review. J. Preclin. Clin. Res..

[220] Yang L., Zhang C., Huang F., Liu J., Zhang Y., Yang C., Ren C., Chu L., Liu B., Liu J. (2020). Triclosan-based supramolecular hydrogels as nanoantibiotics for enhanced antibacterial activity. J. Control Release.

[221] Rodricks J.V., Swenberg J.A., Borzelleca J.F., Maronpot R.R., Shipp A.M. (2010). Triclosan: A critical review of the experimental data and development of margins of safety for consumer products. Crit Rev. Toxicol..

[222] Bhargava H.N., Leonard P.A. (1996). Triclosan: Applications and safety. Am. J. Infect. Control.

[223] Weatherly L.M., Shim J., Hashmi H.N., Kennedy R.H., Hess S.T., Gosse J.A. (2016). Antimicrobial agent triclosan is a proton ionophore uncoupler of mitochondria in living rat and human mast cells and in primary human keratinocytes. J. Appl. Toxicol..

[224] Weatherly L.M., Nelson A.J., Shim J., Riitano A.M., Gerson E.D., Hart A.J., de Juan-Sanz J., Ryan T.A., Sher R., Hess S.T. (2018). Antimicrobial agent triclosan disrupts mitochondrial structure, revealed by super-resolution microscopy, and inhibits mast cell signaling via calcium modulation. Toxicol. Appl. Pharmacol..

[225] Sangroula S., Vasquez A.Y.B., Raut P., Obeng B., Shim J.K., Bagley G.D., West B.E., Burnell J.E., Kinney M.S., Potts C.M. (2020). Triclosan disrupts immune cell function by depressing Ca^2+^ influx following acidification of the cytoplasm. Toxicol. Appl. Pharmacol..

[226] González-Pleiter M., Rioboo C., Reguera M., Abreu I., Leganés F., Cid Á., Fernández-Piñas F. (2017). Calcium mediates the cellular response of *Chlamydomonas reinhardtii* to the emerging aquatic pollutant triclosan. Aquat. Toxicol..

[227] Pullaguri N., Kagoo A.R., Bhargava A. (2022). New insights into inhibitory nature of triclosan on acetylcholinesterase activity. Toxicology.

[228] Zhao C., Xie R., Qian Q., Yan J., Wang H., Wang X. (2022). Triclosan induced zebrafish immunotoxicity by targeting miR-19a and its gene socs3b to activate IL-6/STAT3 signaling pathway. Sci. Total Environ..

[229] Kwon J.T., Yang Y.S., Kang M.S., Seo G.B., Lee D.H., Yang M.J., Shim I., Kim H.M., Kim P., Choi K. (2013). Pulmonary toxicity screening of triclosan in rats after intratracheal instillation. J. Toxicol. Sci..

[230] Yang Y.S., Kwon J.T., Shim I., Kim H.M., Kim P., Kim J.C., Lee K. (2015). Evaluation of toxicity to triclosan in rats following 28 days of exposure to aerosol inhalation. Reg. Toxicol. Pharmacol..

[231] Liang Y., Zhang H., Cai Z. (2021). New insights into the cellular mechanism of triclosan-induced dermal toxicity from a combined metabolomic and lipidomic approach. Sci. Total Environ..

[232] Zhang M., Zhu R., Zhang L. (2020). Triclosan stimulates human vascular endothelial cell injury via repression of the PI3K/Akt/mTOR axis. Chemosphere.

[233] Yoon D.S., Choi Y., Cha D.S., Zhang P., Choi S.M., Alfhili M.A., Polli J.R., Pendergrass D., Taki F.A., Kapalavavi B. (2017). Triclosan disrupts SKN-1/Nrf2-mediated oxidative stress response in *C. elegans* and human mesenchymal stem cells. Sci. Rep..

[234] Bao S., He C., Ku P., Xie M., Lin J., Lu S., Nie X. (2021). Effects of triclosan on the RedoximiRs/Sirtuin/Nrf2/ARE signaling pathway in mosquitofish (*Gambusia affinis*). Aquat. Toxicol..

[235] Hemalatha D., Rangasamy B., Nataraj B., Ramesh M. (2019). Assessment of triclosan impact on enzymatic biomarkers in an Indian major carp, *Catla catla*. J. Basic Appl. Zool..

[236] Zhang Q., Hao L., Hong Y. (2021). Exploring the multilevel effects of triclosan from development, reproduction to behavior using *Drosophila melanogaster*. Sci. Total Environ..

[237] Liu M., Ai W., Sun L., Fang F., Wang X., Chen S., Wang H. (2019). Triclosan-induced liver injury in zebrafish (*Danio rerio*) via regulating MAPK/p53 signaling pathway. Comp. Biochem. Physiol. C Toxicol. Pharmacol..

[238] Sharma S., Dar O.I., Singh K., Kaur A., Faggio C. (2021). Triclosan elicited biochemical and transcriptomic alterations in *Labeo rohita* larvae. Environ. Toxicol. Pharmacol..

[239] Kim J.Y., Yi B.R., Go R.E., Hwang K.A., Nam K.H., Choi K.C. (2014). Methoxychlor and triclosan stimulates ovarian cancer growth by regulating cell cycle- and apoptosis-related genes via an estrogen receptor-dependent pathway. Environ. Toxicol. Pharmacol..

[240] Wang Z., Li X., Klaunig J.E. (2017). Investigation of the mechanism of triclosan induced mouse liver tumors. Regul. Toxicol. Pharm..

[241] Wu M., Zhao G., Zhuang X., Zhang T., Zhang C., Zhang W., Zhang Z. (2018). Triclosan treatment decreased the antitumor effect of sorafenib on hepatocellular carcinoma cells. OncoTarg. Ther..

[242] Kim S.H., Hwang K.A., Shim S.M., Choi K.C. (2015). Growth and migration of LNCaP prostate cancer cells are promoted by triclosan and benzophenone-1 via an androgen receptor signaling pathway. Environ. Toxicol. Pharmacol..

[243] Lee H.-R., Hwang K.-A., Nam K.-H., Kim H.-C., Choi K.-C. (2014). Progression of breast cancer cells was enhanced by endocrine-disrupting chemicals, triclosan and octylphenol, via an estrogen receptor-dependent signaling pathway in cellular and mouse xenograft models. Chem. Res. Toxicol..

[244] Kim S.H., Hwang K.A., Choi K.C. (2016). Treatment with kaempferol suppresses breast cancer cell growth caused by estrogen and triclosan in cellular and xenograft breast cancer models. J. Nutr. Biochem..

[245] Lee G.A., Choi K.C., Hwang K.A. (2018). Treatment with phytoestrogens reversed triclosan and bisphenol A-induced anti-apoptosis in breast cancer cells. Biomol. Ther..

[246] Farasani A., Darbre P.D. (2021). Long-term exposure to triclosan increases migration and invasion of human breast epithelial cells in vitro. J. Appl. Toxicol..

[247] Wu A.H., Franke A.A., Wilkens L.R., Tseng C., Conroy S.M., Li Y., Sangaramoorthy M., Polfus L.M., DeRouen M.C., Caberto C. (2021). Risk of breast cancer and prediagnostic urinary excretion of bisphenol A, triclosan and parabens: The multiethnic cohort study. Int. J. Cancer.

[248] Szychowski K.A., Skóra B., Bar M., Piechowiak T. (2022). Triclosan (TCS) affects the level of DNA methylation in the human oral squamous cell carcinoma (SCC-15) cell line in a nontoxic concentration. Biomed. Pharmacother..

[249] Bilal M., Barceló D., Iqbal H.M. (2020). Persistence, ecological risks, and oxidoreductases-assisted biocatalytic removal of triclosan from the aquatic environment. Sci. Total Environ..

[250] Solá-Gutiérrez C., Schröder S., San-Román M.F., Ortiz I. (2020). Critical review on the mechanistic photolytic and photocatalytic degradation of triclosan. J. Environ. Manag..

[251] Balakrishnan P., Mohan S. (2021). Treatment of triclosan through enhanced microbial biodegradation. J. Hazard. Mater..

[252] Hu B., Hu S., Vymazal J., Chen Z. (2022). Application of arbuscular mycorrhizal fungi for pharmaceuticals and personal care productions removal in constructed wetlands with different substrate. J. Clean. Product..

[253] Awad A.M., Shaikh S.M.R., Jalab R., Gulied M.H., Nasser M.S., Benamor A., Adham S. (2019). Adsorption of organic pollutants by natural and modified clays: A comprehensive review. Sep. Purif. Technol..

[254] Vidovix T.B., Januário E.F.D., Araújo M.F., Bergamasco R., Vieira A.M.S. (2022). Investigation of two new low-cost adsorbents functionalized with magnetic nanoparticles for the efficient removal of triclosan and a synthetic mixture. Environ. Sci. Pollut. Res..

[255] Hua Y., Dai X., Xu Y., Xing G., Liu H., Lu T., Chen Y., Zhang Y. (2022). Drug repositioning: Progress and challenges in drug discovery for various diseases. Eur. J. Med. Chem..

[256] Catalano A., Iacopetta D., Pellegrino M., Aquaro S., Franchini C., Sinicropi M.S. (2021). Diarylureas: Repositioning from antitumor to antimicrobials or multi-target agents against new pandemics. Antibiotics.

[257] Catalano A., Iacopetta D., Ceramella J., Mariconda A., Rosano C., Scumaci D., Saturnino C., Longo P., Sinicropi M.S. The new challenges for the treatment of triple-negative breast cancer. Appl. Sci..

[258] Sadowski M.C., Pouwer R.H., Gunter J.H., Lubik A.A., Quinn R.J., Nelson C.C. (2014). The fatty acid synthase inhibitor triclosan: Repurposing an anti-microbial agent for targeting prostate cancer. Oncotarget.

[259] Bahmad H.F., Demus T., Moubarak M.M., Daher D., Alvarez Moreno J.C., Polit F., Lopea O., Mehre A., Abou-Kheir W., Nieder A.M. (2022). Overcoming drug resistance in advanced prostate cancer by drug repurposing. Med. Sci..

[260] Mesquita J.T., Romanelli M.M., de Melo Trinconi Trinconi C.M., Guerra J.M., Taniwaki N.N., Uliana S.R.B., Reimaão J.Q., Tempone A.G. (2022). Repurposing topical triclosan for cutaneous leishmaniasis: Preclinical efficacy in a murine *Leishmania* (L.) *amazonensis* model. Drug Develop. Res..

[261] Otero E., García E., Palacios G., Yepes L.M., Carda M., Agut R., Vélez I.D., Cardona W.I., Robledo S.M. (2017). Triclosan-caffeic acid hybrids: Synthesis, leishmanicidal, trypanocidal and cytotoxic activities. Eur. J. Med. Chem..

[262] Duarte S.S., de Moura R.O., da Silva P.M. (2018). Effect of antiprotozoal molecules on hypnospores of Perkinsus spp. parasite. Expe. Parasitol..

[263] Vergara S., Carda M., Agut R., Yepes L.M., Velez I.D., Robledo S.M., Galeano W.C. (2017). Synthesis, antiprotozoal activity and cytotoxicityinU-937macrophages of triclosan–hydrazone hybrids. Med. Chem. Res..

